# Recurrent allopolyploidizations diversify ecophysiological traits in marsh orchids (*Dactylorhiza majalis* s.l.)

**DOI:** 10.1111/mec.17070

**Published:** 2023-07-15

**Authors:** Thomas M. Wolfe, Francisco Balao, Emiliano Trucchi, Gert Bachmann, Wenjia Gu, Juliane Baar, Mikael Hedrén, Wolfram Weckwerth, Andrew R. Leitch, Ovidiu Paun

**Affiliations:** ^1^ Department of Botany and Biodiversity Research University of Vienna Vienna Austria; ^2^ Vienna Graduate School of Population Genetics Vienna Austria; ^3^ Department of Forest and Soil Sciences University of Natural Resources and Life Sciences Vienna Austria; ^4^ Departamento de Biologia Vegetal y Ecologia University of Seville Sevilla Spain; ^5^ Marche Polytechnic University Ancona Italy; ^6^ Department of Functional and Evolutionary Ecology, Molecular Systems Biology (MOSYS) University of Vienna Vienna Austria; ^7^ School of Biological and Chemical Sciences Queen Mary University of London London UK; ^8^ Department of Biology Lund University Lund Sweden; ^9^ Vienna Metabolomics Center (VIME) University of Vienna Vienna Austria

**Keywords:** allopolyploidy, *Dactylorhiza*, differential expression, ecological differentiation, photosynthesis, soil chemistry

## Abstract

Whole‐genome duplication has shaped the evolution of angiosperms and other organisms, and is important for many crops. Structural reorganization of chromosomes and repatterning of gene expression are frequently observed in allopolyploids, with physiological and ecological consequences. Recurrent origins from different parental populations are widespread among polyploids, resulting in an array of lineages that provide excellent models to uncover mechanisms of adaptation to divergent environments in early phases of polyploid evolution. We integrate here transcriptomic and ecophysiological comparative studies to show that sibling allopolyploid marsh orchid species (*Dactylorhiza*, Orchidaceae) occur in different habitats (low nutrient fens vs. meadows with mesic soils) and are characterized by a complex suite of intertwined, pronounced ecophysiological differences between them. We uncover distinct features in leaf elemental chemistry, light‐harvesting, photoprotection, nutrient transport and stomata activity of the two sibling allopolyploids, which appear to match their specific ecologies, in particular soil chemistry differences at their native sites. We argue that the phenotypic divergence between the sibling allopolyploids has a clear genetic basis, generating ecological barriers that maintain distinct, independent lineages, despite pervasive interspecific gene flow. This suggests that recurrent origins of polyploids bring about a long‐term potential to trigger and maintain functional and ecological diversity in marsh orchids and other groups.

## 1 INTRODUCTION

Whole‐genome duplication is a central force in evolution (e.g. Van de Peer et al., 2017), and ploidy increase has been estimated to be directly associated with about one in seven angiosperm speciation events (Wood et al., 2009). In animals, polyploidy is rarer, but multiple cases of currently polyploid insects, fishes, amphibians and reptiles are known (Otto & Whitton, 2000). Moreover, genomic evidence suggests all vertebrates, flowering plants and some fungi descended from polyploid ancestors (Otto, 2007; Van de Peer et al., 2017). Polyploidy merges multiple entire genomes in one nucleus, and if polyploids are derived from divergent species (as for allopolyploids), or even from distinct individuals, it can trigger a range of transgressive transcriptomic responses and trans‐acting effects (e.g. Adams & Wendel, 2005; Burns et al., 2021). Polyploids are often reproductively isolated from diploid relatives, causing population density‐dependent fitness costs known as minority cytotype exclusion (Husband, 2000; Levin, 2002). To establish and spread, polyploids are believed to benefit from certain reproductive characteristics, such as assortative mating and a breakdown of self‐incompatibility (Fowler & Levin, 1984, 2016; Soltis et al., 2014).

Polyploidy appears to have significant physiological and ecological consequences, which may lead to establishment in divergent environments that would otherwise be considered stressful for the diploid parents (Novikova et al., 2018; Van de Peer et al., 2017). Furthermore, palaeopolyploidy events tend to cluster around past periods of unstable conditions, including mass extinction events, which also suggests that stressful environments could systematically induce higher rates of polyploidy (e.g. Lohaus & Van de Peer, 2016). Novel ecological properties may arise in early‐generation polyploids as a by‐product of large‐ and small‐scale adjustments of genome organization and gene regulation that can occur with polyploidy, which with meiotic segregation can lead to transgressive characters, especially in allopolyploids (Paun et al., 2007). Several lines of evidence suggest that polyploids can show novel morphological and physiological characteristics compared with their lower ploidy relatives, with indirect consequences on complex ecological interactions, even between different trophic levels (Bomblies, 2020; Ramsey, 2011; Segraves, 2017).

Information on ecophysiological consequences of polyploidy is currently limited (Scheriau et al., 2017; Segraves, 2017; te Beest et al., 2012). Genome multiplication is expected to change the scaling between nucleus and other cellular components, and positive relationships are generally observed between genome size and cell size, guard cell length and epidermal cell area, whereas a negative relationship with stomatal density is observed (Beaulieu et al., 2008; Doyle & Coate, 2019). A main ecophysiological implication of polyploidy is thought to be an increased nutrient requirement for nitrate and phosphate, necessary for DNA replication and chromatin packing in polyploids compared to diploids, especially in habitats with limited nutrient resources (Glennon et al., 2014; Guignard et al., 2016, 2017; Jeyasingh et al., 2015; Ramsey, 2011). System‐specific physiological consequences of polyploidy identified through comparisons between polyploids and related diploids have also been shown, for example, increased salt tolerance (Chao et al., 2013), alterations in water‐use efficiency (Garbutt & Bazzaz, 1983) and in photosynthetic activity (e.g. Coate et al., 2012). Such outcomes may allow neopolyploids to range more broadly over the adaptive landscape and access adaptive peaks that may otherwise be unattainable through incremental changes at the diploid level (Wang et al., 2022). Established polyploids can exhibit larger distributions than their lower ploidy progenitors, especially across habitats that feature less interspecific competition, for example, previously glaciated regions (Novikova et al., 2018; Rice et al., 2019; Stebbins, 1971). Disentangling the ecophysiological implications of polyploidization in a natural context, relative to adaptation during later evolution, is important for understanding how polyploids spread across niches and their effects on biodiversity (Abbott et al., 2013; Jurado et al., 2019; Soltis et al., 2010).

Recurrence in polyploidization is commonly observed during polyploid evolution (Soltis & Soltis, 1999). Examples include up to 20 independent origins of the allopolyploid *Tragopogon miscellus*, 13 origins for *Draba norvegica* and 46 for tetraploid *Galax urceola* (Segraves, 2017). Within the same population, recurrent formation of polyploids from different parental genotypes can greatly enhance genetic diversity in the nascent polyploid (Soltis et al., 2010; Soltis & Soltis, 1999). However, independent whole‐genome duplications can happen in different ecological contexts too, potentially leading to local adaptation and the formation of polyploid species with different evolutionary trajectories (Jurado et al., 2019). Subsequent gene flow, independent assortment and recombination between polyploids can produce additional genetic variation, and it is unclear to what extent and in which conditions such sibling polyploids can maintain distinctiveness in the face of gene flow (Novikova et al., 2020; Paun et al., 2007; Soltis et al., 2010).

**FIGURE 1:**
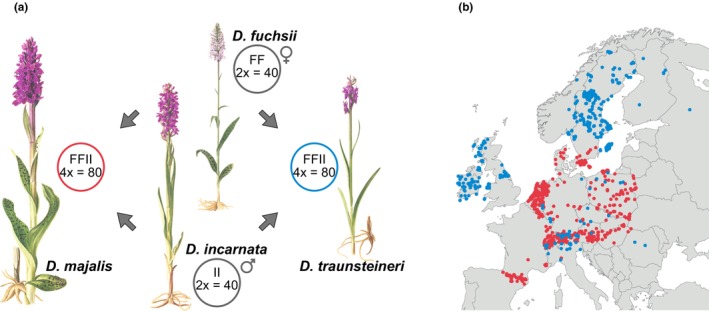
(a) The sibling allotetraploids *Dactylorhiza majalis* and *D*. *traunsteineri* have each originated from allopolyploidizations involving the diploid *D*. *incarnata* acting as the paternal parent and *D*. *fuchsii* as the maternal. The bubbles give the chromosome number and genome configuration of each species. Plant illustrations modified from Nelson (1976). (b) Curated *D*. *majalis* (red) and *D*. *traunsteineri* localities (blue) extracted from GBIF (accessed February 2018).

In this study, we focus on the ecophysiological diversity released by recurrent allopolyploidization events in European marsh orchids (*Dactylorhiza*) in order to understand the context that allows occupation of diverse environments, potentially leading to the evolution and maintenance of distinct allopolyploid species. We investigate two sibling allopolyploids, *Dactylorhiza majalis* and *D*. *traunsteineri* that are estimated to have formed at different times in the recent half of the Quaternary (Brandrud et al., 2020; Hawranek, 2021). They arose from independent, but unidirectional allopolyploidization between *D*. *fuchsii* (as maternal genome) and *D*. *incarnata* (Figure 1a; Brandrud et al., 2020; Pillon et al., 2007). With DIY ABC (Cornuet et al., 2014) and genome‐wide RADseq data, the maximum age of the sibling allopolyploids has been estimated as c. 1730 generations for *D*. *majalis* and *c*. 920 generations for *D*. *traunsteineri* (Brandrud, 2019). However, based on the same data and coalescent inference, Hawranek (2021) estimated a maximum age of c. 104,000 generations for *D*. *majalis* and of c. 74,000 generations for *D*. *traunsteineri*. Apart from applying different demographic inference frameworks, the two estimates used different approaches to separate the homoeologs of the allopolyploids. Due to the considerable difference in the age estimates, further studies are required to refine and validate these estimates. Independent on their exact age, it is clear that *D*. *majalis* is older (Brandrud et al., 2020) and has survived in one or few neighbouring refugia in Central Europe during the last ice age (Balao et al., 2016). *Dactylorhiza traunsteineri* is in turn younger and more heterogeneous (Balao et al., 2016; Brandrud et al., 2020). It occupies almost exclusively previously glaciated ranges, suggesting an origin around the last glacial cycle. However, its regional mosaic of plastid DNA variants, some not found in the present‐day representatives of its diploid parents, could support a pre‐last glacial origin also for *D*. *traunsteineri* (Nordström & Hedrén, 2008, 2009; Pillon et al., 2007). Since their origin, both allopolyploids have experienced a considerable increase in genome size, mainly driven by a putative tandem repeat (Eriksson et al., 2022). However, the repetitive landscapes of either allopolyploid do not generally show signals of a major genomic shock following allopolyploidization.

Each of these allotetraploids occupies wide distributions across Europe. *Dactylorhiza majalis* ranges from the Pyrenees to southern Scandinavia, whereas its younger sibling *D*. *traunsteineri* has a more disjunct distribution around previously glaciated areas: in the British Isles, in northern Europe, and also in the Alps where it can grow in sympatry with *D*. *majalis* (Figure 1b). Both allopolyploids are pollinated through food deceit by naïve bumblebees, which can promote interspecific cross‐pollination (Hedrén & Nordström, 2018). Indeed, based on a wide range of genetic data, frequent and ongoing gene flow has been documented between the two allotetraploid species in the Alps, but with pervasive traces affecting also populations outside strictly sympatric localities (Balao et al., 2016; Brandrud et al., 2020; Hawranek, 2021; Hedrén et al., 2008). A well‐documented example of a strongly admixed population is the type locality of *D*. *traunsteineri* close to Kitzbuhel, Austria (i.e. locality ALP9 in Balao et al., 2016 and, respectively, at8 in Hawranek, 2021), whose individuals exhibit characteristic morphological traits, but generally contain a large portion of *D*. *majalis* alleles.

The two sibling allopolyploid species have been suggested to have distinctive ecologies, even at localities where the two allopolyploids are close neighbours (Paun et al., 2011). However, no quantitative data are available on the type and magnitude of ecological differences. *Dactylorhiza majalis* is represented by robust, broad‐leaved plants with large inflorescences and typically grows in mesic to moist meadows, whereas *D*. *traunsteineri* is slender with narrow leaves and a few‐flowered inflorescence, and it generally occurs in marshes and fens with a continuous influx of underground water; both species can benefit from mowing or grazing (Djordjević et al., 2016; Janečková et al., 2006; Sletvold et al., 2010; Wotavová et al., 2004). *Dactylorhiza traunsteineri* is commonly thought to be a poor competitor and tends to grow in sparse vegetation, whereas *D*. *majalis* generally occupies denser communities where there are more resources (Paun et al., 2010). As established, widespread species (Figure 1b), these sibling allopolyploids will have gone through the filtering effects of natural selection.

Using these sibling marsh orchids, we present a multilevel, integrated investigation of physiology, ecology and gene expression to understand the phenotypic differentiation among them, and begin to disentangle the basis of their adaptation to divergent habitats. We further study differential gene expression in an outdoors common garden experiment, in order to understand the molecular context that allows the two allopolyploids to occupy distinct niches and to maintain phenotypic distinctiveness, despite pervasive gene flow (Balao et al., 2016; Hawranek, 2021).

## 2 MATERIALS AND METHODS

### 2.1 Soil and leaf elemental analyses

We first quantified 18 distinct soil elemental components (N–NO_3_, N–NH_4_, Al, Ca, Cd, Cr, Cu, Fe, K, Mg, Mn, Mo, Na, Ni, P, Pb, S, Zn) and three soil features (pH, N%, C%) at 18 different localities across Europe (Table S1). The soil samples were collected in spring 2017 for nine localities of *D*. *majalis* (five from the Alps, two from the Pyrenees and two from Scandinavia), and nine localities of *D*. *traunsteineri* (three from the Alps, three from the Britain and three from Scandinavia). To obtain an accurate characterization, soil measurements were performed for each locality for two composite soil samples, each including three subsamples in approximately equal proportion. The soil was dried, and the elemental composition was measured using a Truspec CN Elemental analyser (LECO Corporation). Available nitrate and ammonium were extracted in 1 M KCl and measured on a plate reader photometer (Bio‐Rad). In addition, a Direct Soil pH Meter HI99121 (Hanna Instruments) was used to measure soil pH at c. 7 cm depth in the close proximity of plants at 22 European localities in multiple years from 2010 to 2015 (Table S1). Significance of distribution differences for soil characteristics between the two allotetraploids was tested using permutation tests with the *permTS* function in the R package ‘perm’ (Fay & Shaw, 2010).

To test if the soil elemental profiles specific for each allotetraploid translate to a differential chemistry in the plant, we further analysed leaf chemical content (i.e. C, N and P) of wild plants at 19 localities across Europe (Table S1). Leaf tissue was collected in silica gel at nine *D*. *majalis* localities (five from the Alps, two from the Pyrenees and two from Scandinavia) and at 10 *D*. *traunsteineri* populations (three from the Alps, five from Britain and two from Scandinavia). To measure N and C in the leaf tissue, 1–2 mg oven‐dried ground sample was mixed with a urea solution and the mixtures were quantified on a mass spectrometer (Integra 2). One blank and two case‐Rs as reference were also loaded for every 10 samples. To measure P, 0.8–2 mg oven‐dried, ground tissue was mixed with 0.15 g potassium persulphate and 1 mL 1 N sulphuric acid and extracted at 121°C for 40 min. The P quantification was finally performed on a Skalar machine (Segment Flow Analyser, San++, Skalar Analytical B.V.). Significance was assessed using permutation tests as explained above.

### 2.2 Macro‐environmental climatic niche characterization

We explored if macro‐environmental differences exist between the two allotetraploids. Occurrence information for the two allotetraploid species was collected from Global Biodiversity Information Facility (GBIF.org., 2020; https://www.gbif.org): A total of 958 *D*. *majalis* and 631 *D*. *traunsteineri* localities were initially extracted using the package ‘rgbif’ (Chamberlain et al., 2017) in R software v.3.4.2. The localities were manually curated based on expert knowledge, duplicates were removed and locally dense sampling was reduced by thinning the records to one per 10 km^2^ grid cell size, retaining 298 localities for *D*. *majalis* and 393 localities for *D*. *traunsteineri* (Figure 1b). We further extracted data for 36 environmental variables from the CHELSA database (Karger et al., 2017) and the ENVIREM database (Title & Bemmels, 2018) for these localities at ~1 km^2^ resolution. We used the variance inflation factor (VIF; threshold of 10) to remove highly correlated environmental variables in the data set. This retained only 15 of the 36 environmental variables, summarizing isothermality (BIO3), temperature seasonality (BIO4), mean temperature of the wettest quarter (BIO8), mean temperature of the driest quarter (BIO9), precipitation seasonality (BIO15), precipitation of warmest quarter (BIO18), precipitation of coldest quarter (BIO19), index of the degree of water deficit below water need (aridityIndex), sum of mean monthly temperature for months with mean temperature greater than 5°C multiplied by number of days (growingDegDays5), minimum temperature of the warmest month (minTempWarmest), mean monthly potential evapotranspiration (PET) of the coldest quarter (PETColdestQuarter), mean monthly PET of the driest quarter (PETDriestQuarter), monthly variability in PET (PETseasonality), mean monthly PET of the wettest quarter (PETWettestQuarter) and topographic wetness index (topoWet).

To compare the macro‐environmental niche of the sibling allotetraploids, we calculated the kernel‐smoothed densities of all occurrence data along environmental axes from a principal component analysis (PCA‐env; Broennimann et al., 2012). For background, we extracted environmental data from 3000 spatially random localities within a buffer of 100 km of land around the occurrence localities. Then, we performed a niche equivalence test (Broennimann et al., 2012) to compare the observed environmental niche overlap (Schoener's D‐statistic) with a null distribution based on 1000 replicates and an environmental grid resolution of 500 × 500 pixels in the R package *ecospat* v. 3.2. (Di Cola et al., 2017). In the niche divergence test, we used Student's *t*‐tests to evaluate the differences in PCA scores between the two allotetraploids.

### 2.3 Photosynthesis and gas exchange

To complement the transcriptomic and ecological investigations, we phenotyped accessions of both allopolyploids in the wild for leaf photosynthetic parameters using a MultispeQ v.1.0 (Kuhlgert et al., 2016). For a total of 38 individuals of *D*. *traunsteineri* and 20 individuals of *D*. *majalis*, we quantified photosynthetic parameters (i.e. SPAD relative chlorophyll; LEF, linear electron flow; PAR, photosynthetically active radiation (light intensity); Phi2, quantum yield of Photosystem II; NPQt, non‐photochemical quenching; PhiNPQ, ratio of incoming light that goes towards non‐photochemical quenching), against momentary air temperature and humidity. We took two replicate measures on different leaves for each accession in the field at two localities in the Alps, where *D*. *majalis* and *D*. *traunsteineri* are within a few hundred metres of each other (i.e. ALP9, ALP13; Table S1) and one population where only *D*. *traunsteineri* grows (ALP8). Over a period of three sunny end‐of‐May days, we randomized as much as possible the effect of the time of the day by alternating measurements between species.

Because we assumed that our data could be clustered or correlated due to our experimental set‐up, we fitted the photosynthesis parameters measured on the MultispeQ device with mixed linear models implemented in the *lmer* function of the lme4 R package (Bates et al., 2015). We first tested the normality of the data using the *descdist* function of the fitdistrplus R package (Delignette‐Muller & Dutang, 2015), and then fitted a null model with each measured photosynthesis variable as outcome and individuals, time of the day and date of the measurements as random effect explanatory factors. This gave us a null distribution for each photosynthesis outcome against which we tested the full distribution with the species factor as an explanatory variable fixed factor. We tested for the significance of species effect on the fitted data using the likelihood ratio test implemented in the anova R function (Bolker et al., 2009). We considered each photosynthesis response as independent to each other, so we have one model per factor. This might not be ideal for modelling, since different photosynthesis outcome could presumably be correlated. We did not explicitly test for such interactions since we aimed to obtain results that could reflect all facets of photosynthesis and could provide the context for interpreting gene expression and other phenotypic differences between the sibling allopolyploids. The detailed results of the ANOVA test between the null hypothesis distribution as described above, and the full distribution including species and an explanatory factor, can be found in the Table S4.

Finally, we measured leaf stomatal conductance for water and net CO_2_ exchange in two replicates of each species in a controlled lab environment using a portable gas exchange fluorescence system GFS‐3000 (Heinz Walz). During the experiment, the conditions were set to 25°C during day and 23°C in the night, 50% air humidity, 12 h photoperiod at 400 μmoles m^−2^ s^−1^ with a LED simulated sunlight spectrum set to 5800 K, watering the pots daily to field capacity.

### 2.4 Reference genome assembly and annotation

To enable further transcriptomic analyses, we assembled de novo a draft genome for a diploid individual of *D*. *incarnata* from Austria (48°11.6′N, 16°29.0′ E) using 177.6 Gb PacBio long reads (i.e. c. 50.7× coverage). We chose to assemble a representative of the paternal species to the allopolyploids despite its larger genome size compared to the maternal *D*. *fuchsii* (3.6 pg vs. 2.9 pg; Aagaard et al., 2005; Eriksson et al., 2022), because of its marked reduced heterozygosity levels compared to *D*. *fuchsii* (Balao et al., 2017; Brandrud et al., 2020). PacBio library preparation and sequencing of 18 SMRT cells on a Sequel I instrument was performed at the sequencing facility of the Vienna BioCenter Core Facilities (VBCF; https://www.viennabiocenter.org/). PacBio data are available from GenBank BioProject PRJNA934399. The assembly was performed with Canu v.1.8 (Koren et al., 2017) with default settings, including the recommended correctedErrorRate 0.045. Based on the PacBio reads, the raw assembly was scaffolded with SSPACE‐LongRead v.1–1 (Boetzer & Pirovano, 2014) and polished with Arrow v.2.3.3 (available from https://github.com/PacificBiosciences/GenomicConsensus). The genome was finally curated to minimize inclusion of both allelic contigs as haplotigs using minimp2 (Li, 2018) and the Purge Haplotigs pipeline (Roach et al., 2018). The draft *D*. *incarnata* genome v.1.0 (GenBank BioProject PRJNA934399) contains 3413 scaffolds and has an N50 size of 7.4 Mb, recovering a total length of 3.27 Gb. This corresponds to 94.3% of the estimated genome size (1C = 3.6 pg, Aagaard et al., 2005; Eriksson et al., 2022).

Before gene annotation analyses, we generated a custom repeat library for repeat masking using RepeatModeler v.1.0.11 (http://www.repeatmasker.org/RepeatModeler/). De novo TE annotation was performed with RepeatMasker v.4.0.7 (https://www.repeatmasker.org/RepeatMasker/) and the search engine set to NCBI (−e ncbi). This process masked as repeat sequences 83.7% of the genome reference. The genome was structurally annotated ab initio using Augustus (Stanke et al., 2006) and GeneMark‐ET (Lomsadze et al., 2014), as implemented in BRAKER1 v.2.1.0 (Hoff et al., 2016) with the options ‐‐softmasking = 1 ‐‐filterOutShort. Mapped mRNA‐seq data of the same accession were used to improve de novo gene finding. An additional source of extrinsic evidence was single‐copy protein sequences predicted by BUSCO v.3 (Simão et al., 2015) when run on the hard masked genome in genome mode with option ‐‐long. The annotation was further improved using MAKER‐P v.2.31.10 (Campbell et al., 2014) supplying gene models identified using BRAKER1. A transcriptome was assembled using Trinity v.2.4.0 (Haas et al., 2013) based on mRNA‐seq data, and this has been used in MAKER‐P as expressed sequence tags (EST). The annotation used as additional evidence (declared as *altest*) the transcriptome of *Orchis italica* (De Paolo et al., 2014) and protein sequences of *Phalaenopsis equestris* (Cai et al., 2015). Structural annotations identified a total of 52,665 protein‐coding gene models. They were functionally annotated using Blast2GO v.5.2.5 (Götz et al., 2008) and the Viridiplantae database, recovering full annotations for 31,394 genes. Finally, we evaluated the completeness of the genome assembly by searching our gene models against the BUSCO v.3 Liliopsida odb10 data set (Simão et al., 2015). A total of 89.2% of the set of single‐copy conserved BUSCO genes were found within our annotated genes, out of which 15.3% genes were recorded as duplicated.

### 2.5 RNA‐seq analyses

To examine the functional differentiation between the two sibling allotetraploids, we further searched for gene expression differences in a common garden set‐up. We transplanted 11 representative adult *D*. *traunsteineri* plants from nine localities throughout the distribution range and eight adult *D*. *majalis* plants from eight localities (see Figure S1 and Table S1), and grew them in an outdoors common garden setting for 2 years in Vienna, Austria, with the aim of removing carry‐over environmental differences. Every year, these perennial orchids store nutrients into a new tuber to support growth in the following year. Leaf tissue was fixed in RNAlater (Sigma) for all accessions in the morning of 14 May 2014 as quickly as possible and in a similar developmental stage. The samples fixed in RNAlater have been stored at −80°C until processing. The wet lab procedure followed the details given in a complementary study focusing on the diploid parents (Balao et al., 2017). The RNA‐seq libraries were sequenced as directional, 100 bp paired‐end reads with Illumina HiSeq at the Vienna BioCenter Core Facilities (VBCF; https://www.viennabiocenter.org/). The RNA‐seq data (Wolfe et al., 2023) are available from GenBank BioProject PRJNA317244.

Read trimming was performed with Trimmomatic v.0.33 with LEADING:3 TRAILING:3 SLIDINGWINDOW:4:20 MINLEN:50. For differential expression analyses, the quality‐trimmed RNA‐seq reads were mapped to the *D*. *incarnata* v.1.0 reference genome with STAR v.2.5.2a (Dobin et al., 2013) using the gene models annotated. We checked for transcript 5′–3′ coverage biases known to affect RNA‐seq experiments with RSeQC (Wang et al., 2012). No substantial transcript coverage bias in our data was found (Figure S4). The table of counts was obtained using the *featureCounts* function in Rsubread R package using the full transcripts as features for which to count the reads (Liao et al., 2013).

The *RUVs* normalization approach implemented in the RUVseq v.1.8.0 R package (Risso et al., 2014) was applied to the table of counts for removal of technical variance. *RUVs* use technical replicates as controls to perform factor analyses on the count matrix and return a linear model that is used to correct for unwanted variance in differential expression analyses. Three (*k* = 3) factors of unwanted variance were removed as this yielded the best overlap between technical replicates overall; *k* = 1, 2 and 4 were also tested. We checked these normalizations with Relative Log Expression plots (*plotRLE* function) and principal component analyses plots (*plotPCA* function) using the EDAseq v.2.8.0 R package (Risso et al., 2011). We then fitted the set of read counts using the *glmQLFit* function of *EdgeR* v.3.24.3 (Robinson et al., 2010) with a non‐intercepting model for species and normalizing factors obtained from *RUVs* as fixed effect variables (McCarthy et al., 2012). The *glmQLFTest* function was applied to the fitted model and an FDR < 0.05 threshold for differential expression for the a priori species contrast was set.

The genome annotation was then imported into *R* with topGO v.2.26.0 (Alexa & Rahnenfuhrer, 2018) and enrichments between pairs of species were done using a two‐sided Fisher's exact test against the set of retained transcripts with an FDR smaller than 0.05. Visualization and summary of overrepresented GO terms were performed using the REVIGO web program and results were exported into R. We also used the GOplot v.1.0.2 R package (Supek et al., 2011; Walter et al., 2015). In order to have the directionality of the differential expression for the enriched GO terms towards one species or another, we calculated the *z*‐score for each GO category as *z*‐score = (#genes_up_ − #genes_down_)/#genes_total_. The table of counts and scripts used for differential expression analyses is available at https://github.com/twolfe/dactylorhiza.

## 3 RESULTS

### 3.1 The sibling allopolyploid marsh orchids are ecologically distinct

**FIGURE 2:**
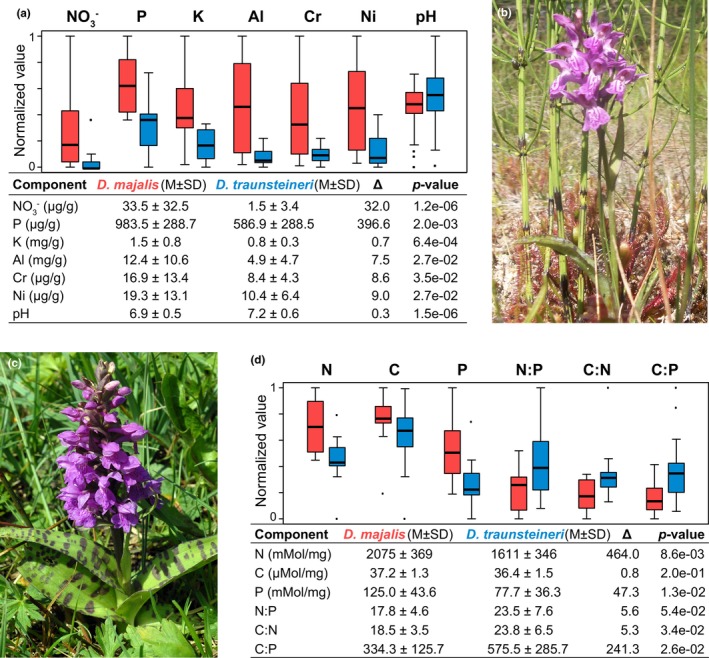
(a) Divergent preference for selected soil characteristics between two sibling *Dactylorhiza* allopolyploids: *D*. *majalis* (red) and *D*. *traunsteineri* (blue). Soil elemental profiling for available nitrate (NO_3_^−^), phosphate (P), potassium (K), aluminium (Al), chromium (Cr), nickel (Ni) and soil pH across multiple European populations. (b) *Dactylorhiza traunsteineri* growing alongside carnivorous sundews (*Drosera* spp.) at a locality on Gotland island, Sweden. (c) *Dactylorhiza majalis*, growing in a typical meadow at a site near Örup, Skåne, Sweden. (d) Leaf elemental profiling for wild individuals of *D*. *majalis* (red) and *D*. *traunsteineri* (blue) at multiple European localities. Data for nitrogen (N), carbon (C), phosphorus (P) and their pairwise ratios in leaf tissues. For (a, d), the data have been normalized to a 0–1 range using feature scaling only for visualization purposes, where *x*_norm_ = (*x* − *x*_min_)/(*x*_max_ − *x*_min_). The tabular form summarizes the raw data: Δ, mean difference; M, average; SD, standard deviation. The *p*‐values are for 1000 permutation tests.

Using soil chemistry analyses and permutation tests, we determined that relative to *D*. *majalis*, *D*. *traunsteineri* (Figure 2b) occurs in soils depleted of several macro‐ and micronutrients (Figure 2a and Table S2), in particular characterized by extremely low levels of available nitrate (NO_3_^−^), but also phosphate (P) and potassium (K). In contrast, *D*. *majalis* (Figure 2c) occurs in richer soils, albeit its populations show in general a wide variance in soil elemental content (Figure 2a). No significant difference was recorded for available ammonium (NH4+) between the specific soils for the two *Dactylorhiza* allotetraploids (Table S2). Soil pH monitored across multiple years across the distribution range was also found to be significantly different, with *D*. *traunsteineri* tending to grow in slightly more alkaline soils than *D*. *majalis* (Figure 2a and Table S2).

Leaf elemental analyses (Figure 2d) showed that relative to *D*. *majalis*, the narrower leaves of *D*. *traunsteineri* (Figures 1 and 2) had significantly lower levels of N and P per weight unit, but not significantly less carbon (C), resulting in higher C:N and C:P ratios.

**FIGURE 3:**
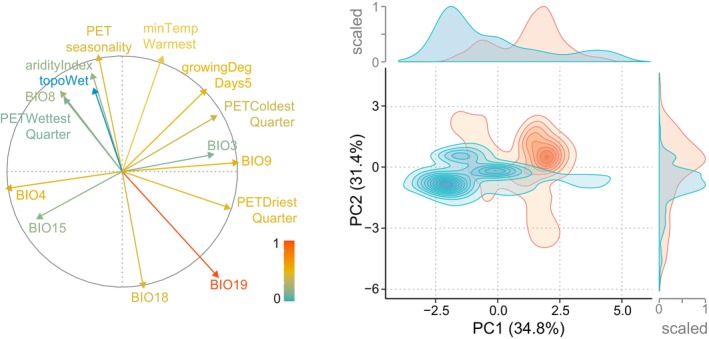
Principal component analysis of no‐collinear macro‐environmental variables performed for GBIF localities and background. The distribution of the selected variables loading on the two main axes is given on the left, with inertia explained by colour of the arrows according to the legend. Bioclim codes are explained in the text. On the right, density contour plots encompass occurrence points of the *D*. *majalis* (red) and *D*. *traunsteineri* (blue) in the 2D environmental space. The occurrence density curves for each axis showed greater environmental divergence in PC1 (Δ = 1.60, *p* < .001) compared to PC2 (Δ = 0.61, *p* < .001).

Although overlapping, the climatic niches for the two allotetraploids were not identical (Niche equivalency test, Schoener's *D* = 0.32, *p* < .001; Figure 3). In particular, temperature seasonality (BIO4), mean temperature of the driest quarter (BIO9), precipitation seasonality (BIO15) and potential evapotranspiration of the driest quarter (PETDriestQuarter) appeared to contribute specificity to the climatic niches of each allotetraploid (Niche divergence test PC1, Student's *t* = 16.444, df = 962.31, *p* < .001; Figure 3). These results are in agreement with *D*. *traunsteineri* generally growing in more northern areas (Figure 1b), but also in wetter habitats than its sibling, *D*. *majalis*.

### 3.2 The sibling allopolyploid marsh orchids are physiologically distinct

**FIGURE 4:**
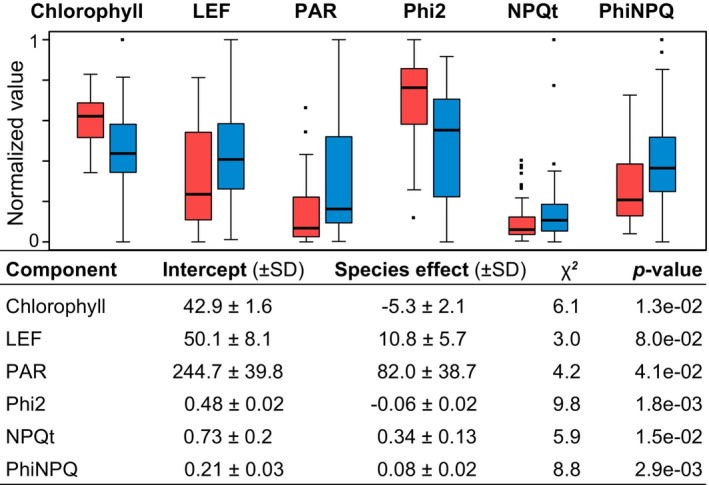
Photosynthetic characteristics for the sibling allotetraploids *D*. *majalis* (red) and *D*. *traunsteineri* (blue) at native localities in the Alps. LEF, linear electron flow; PAR, photosynthetically active radiation (light intensity); Phi2, quantum yield of photosystem II; NPQt, non‐photochemical quenching; PhiNPQ, ratio of incoming light that goes towards non‐photochemical quenching. The boxplots show normalized values only for visualization purposes calculated as in Figure 2. The tabular form gives the details of the likelihood ratio between the null model with time, date and individual measurements as random variables, and the full model with species added to the model as a fixed variable. SD, standard deviation.

Compared to *D*. *majalis*, the narrower leaves of *D*. *traunsteineri* (Figures 1 and 2) showed significantly less relative chlorophyll content in native conditions (Figure 4 and Table S4). *D*. *traunsteineri* used 82.0 ± 38.7 μmol photons $\;m^{-2}\times s^{-1}$ more incoming light (400–700 nm) to drive photosynthesis (PAR), compared to *D*. *majalis*. However, the quantum yield of PSII (Phi2), that is, the ratio of excited electrons that go into the PSII, was significantly lower in *D*. *traunsteineri* than in *D*. *majalis*. Finally, the amount of excess energy (i.e. excited electrons) regulated away from photosynthetic processes in order to reduce damage to the plant (i.e. non‐photochemical quenching, phiNPQ) was significantly higher in *D*. *traunsteineri* (Figure 4 and Table S4). Despite the proximity of the two species at the sympatric localities and our randomized sampling, the MultispeQ measurements uncovered a difference in the ambient conditions around the two allopolyploids. In accordance with its wet and exposed habitats (i.e. low surrounding vegetation), *D*. *traunsteineri* experienced on average 3.5% ± 1.1 (std. error) more ambient humidity (*p* = .0045) and a 1.4 ± 0.4°C higher temperature (*p* = 2e‐10) than *D*. *majalis*.

**FIGURE 5:**
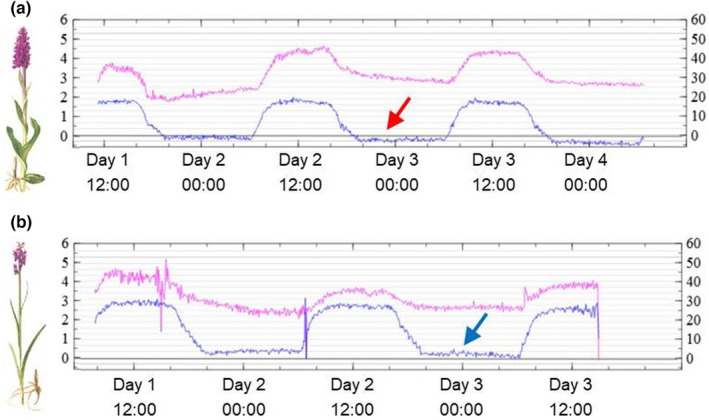
Examples of leaf stomatal conductance for water vapour (pink line, mmol m^−2^ s^−1^ on the right‐side Y‐axes) and net CO_2_ assimilation rate (blue line, μmoles m^−2^ s^−1^ on the left‐side Y‐axes) for an individual of *D*. *majalis* (a) and one of *D*. *traunsteineri* (b). The levels of stomatal conductance and CO_2_ exchange fluctuate with a 12‐h day/night cycle. Stomatal conductance is lower in *D*. *traunsteineri* which shows that its leaves do not open stomata as much as *D*. *majalis*, resulting in less water loss (higher water use efficiency) for nutrient acquisition via xylem. At night, the net CO_2_ exchange is slightly negative for *D*. *majalis* (red arrow) meaning that it releases more CO_2_ through respiration during nighttime, in contrast to *D*. *traunsteineri* (blue arrow), which also features 50% higher CO_2_ assimilation rate during daytime. Plant illustrations modified from Nelson (1976).

Regarding leaf stomatal conductance for water and net CO_2_ exchange, we found that both allotetraploids maintained their stomata open at night, and hence, gas exchange through stomata occurred throughout the night (Figure 5), likely using evapotranspiration to maintain xylem transport to accommodate for nutrient scarcity. However, during the night, *D*. *majalis* showed a negative net CO_2_ (i.e. a release of CO_2_) in contrast to *D*. *traunsteineri*. In addition, during daytime *D*. *traunsteineri* transpires c. 20% less water, and this higher water‐usage efficiency complements a c. 50% higher CO_2_ net assimilation rate compared to *D*. *majalis*. What this means for the enzyme apparatus and the light to energy conversion of the species needs to be elucidated in future research. As the transcriptomic aspects and gas exchange results were obtained in a common garden, it appears likely that these physiological features are genetically encoded.

### 3.3 Distinct ecophysiologies are associated with a complex suite of transcriptomic differences

**FIGURE 6:**
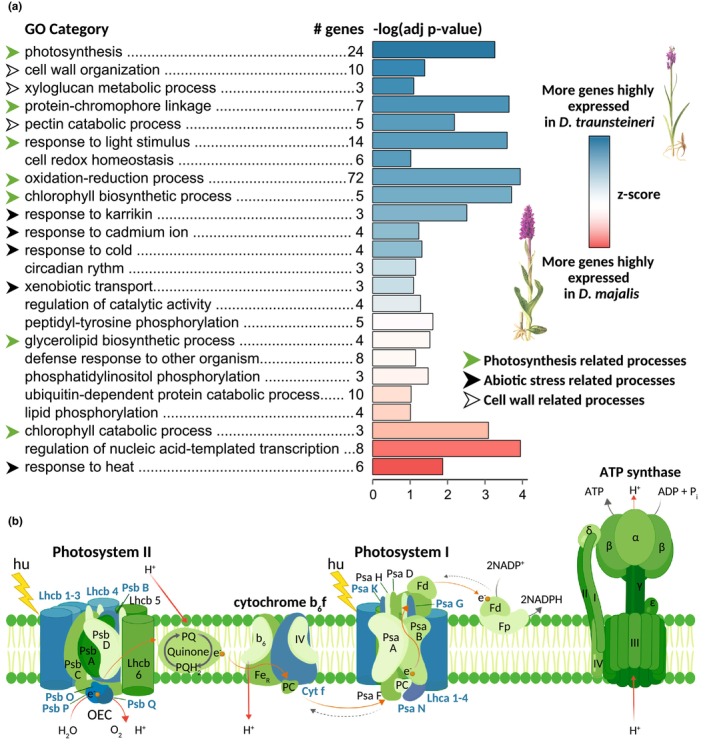
(a) Summary of the enriched gene ontology (GO) categories and the number of differentially expressed genes in each of the GO terms. The length of the bars shows the ‐log(adjusted *p*‐value) of the category's enrichment, and the bar's colour illustrates the z‐score for the category according to the legend. Blue is for categories where there are more genes highly expressed in *D*. *traunsteineri*, and red is for categories where there are more genes highly expressed in *D*. *majalis*. Pale colours show that the respective categories have a more balanced ratio of genes that have increased expression in either species. Plant illustrations modified from Nelson (1976). (b) A summary of photosynthetic reactions drawn with BioRender (https://biorender.com), indicating in blue shades, the components higher expressed in *D*. *traunsteineri* compared to *D*. *majalis*. Fd, ferredoxin; OCE, oxygen‐evolving complex; PC, plastocyanin.

Principal component analysis (PCA, Figure S2) based on the levels of gene expression produced partly overlapping clusters of the two allopolyploids, and revealed some geographic signal, in particular within *D*. *traunsteineri*, likely reflecting its disjunct distribution (Figure 1b). Differential gene expression tests revealed that 316 genes (2.5% of those retained after count‐per‐million‐based filtering) were significantly higher expressed in *D*. *majalis*, and 343 (2.7% of retained genes) significantly higher expressed in *D*. *traunsteineri* (Figure 6a and Figure S3). Photosynthesis (GO:0015979) and related processes were by far the most affected by differential expression between *D*. *majalis* and *D*. *traunsteineri* (Table S3). In particular, relative to *D*. *majalis*, *D*. *traunsteineri* shows an increased expression of the Lhca1–Lhca4 and Lhcb1–Lhcb4 antenna proteins that are part of the light‐harvesting complex (LHC), which captures and delivers excitation to photosystems (Figure 6b; Li et al., 2004). Relatively higher expressed in *D*. *traunsteineri* are also several components of the oxygen‐evolving complex of the Photosystem II (PSII; Figure 6b) that are responsible for catalysing the cleavage of water to oxygen, protons and electrons (Raymond & Blankenship, 2004). *Dactylorhiza traunsteineri* has also higher expression in the Photosystem I (PSI) reaction centres V (psaG), X (psaK), N (psaN) and O (psaO) (Figure 6b), which are mediating the primary function of the PSI (i.e. electron transfer from plastocyanin to ferredoxin; Jensen et al., 2004). Notably, 10.9% of the differentially expressed genes (i.e. mostly higher expressed in *D*. *traunsteineri*) are involved in oxidation–reduction, a process that also shows one of the strongest over‐representations in the enrichment test (Figure 6a). Also related to photosynthesis, several genes linked to protein–chromophore linkage and in the response to light stimulus are higher expressed in *D*. *traunsteineri* compared to *D*. *majalis* (Figure 4a). Chlorophyll biosynthesis processes appeared to be higher expressed in *D*. *traunsteineri*, whereas chlorophyll catabolic processes higher expressed in D. majalis. Several processes related to cell wall organization are found to be in general higher expressed in *D*. *traunsteineri*, including some linked to pectin catabolic processes and xyloglucan metabolism. Finally, some processes related to response to abiotic stress have been identified to be differentially expressed between the two species, including six genes involved in the response to heat and four linked to the response to cold.

## 4 DISCUSSION

Although the sibling species *D*. *majalis* and *D*. *traunsteineri* arose from recent unidirectional allopolyploidization events (Figure 1a; Brandrud et al., 2020; Hawranek, 2021; Pillon et al., 2007) and share part of their distribution area in Central Europe (Figure 1b), our investigations uncovered major and intertwined ecological, physiological and transcriptomic differences between them, despite not estimating directly the fitness of either species in the alternative habitats. In particular, whereas *D*. *majalis* generally occurs in mesic meadows, *D*. *traunsteineri* is found in environments with extremely poor mineral nutrition and low competition (Figure 2a; Paun et al., 2010), for example, often occurring alongside carnivorous plants such as sundews (*Drosera* spp.; Figure 2b). Importantly, the niches of the sibling allopolyploids are characterized by a marked difference in available NO_3_^−^ content (Figure 2a), but not NH_4_^+^. Ammonium can provide a source of nitrogen (N) for plants, particularly in waterlogged soils, but the metabolic processes necessary to assimilate this form of N are different from a nitrate‐based metabolism (Xu et al., 2012). In contrast to NO_3_^−^ which is processed in the leaves, NH_4_^+^ is metabolized directly in the roots, a process that requires sugars which have to be transported from leaves to roots. Ammonium uptake can affect plant foraging of other nutrients, in particular, K, Ca and Mg.

The specific nutrient‐poor soils preferred by *D*. *traunsteineri* (Figure 2a) and the nitrogen deficiency quantified in its leaves in natural conditions (Figure 2d) are likely to be responsible for its low relative chlorophyll content (Figure 4), as nitrogen is required for chlorophyll synthesis (e.g. Allison et al., 1997). We observed a relatively higher expression of genes involved in light harvesting (Figure 6) in *D*. *traunsteineri* compared to *D*. *majalis* in an outdoors common garden setting, and it is likely that chlorophyll deficiency became ameliorated by an increased activity of light harvesting proteins. Such alterations to light‐harvesting complexes may make regulation of captured light more difficult, resulting in a higher linear electron flow, and respectively, non‐photochemical quenching of excited electrons for *D*. *traunsteineri* (Figure 4). The increased phiNPQ in *D*. *traunsteineri* compared to *D*. *majalis* as measured in the field may be reflected in its relatively higher expression of oxidation–reduction processes (strongly enriched in differential expression tests in the outdoors common garden; Figure 6a), with a role in coping with residual oxygen radicals. This suggests that *D*. *traunsteineri* needs to implement a stronger photoprotective strategy to regulate its photosynthetic activity via quenching, potentially due to the limiting amount of chlorophyll, itself likely reflecting a deficiency of nitrate and other nutrients at those respective sites (Demmig‐Adams & Adams, 2006; Lopez‐Jurado et al., 2020). However, in spite of its lower mineral soil composition and respectively its decreased N and P leaf content, *D*. *traunsteineri* has an increased overall photosynthetic efficiency to fix C, compared to *D*. *majalis*, as indicated by the higher leaf ratio of C to N, and of C to P.

Previous genetic studies (Balao et al., 2016; Brandrud et al., 2020; Hedrén et al., 2008; Hawranek, 2021) have reported pervasive gene flow between the two allotetraploids and a relatively high rate of backcrossing across their distribution range. The gene expression data support the hypothesis of extensive hybridization in sympatry, as the PCA plot based on gene expression shows the accessions of both allopolyploids from the Alps are intermingled (accessions shown with squares in Figure S2). Yet despite ongoing hybridization, the morphological differences between *D*. *majalis* and *D*. *traunsteineri* are clear, even for individuals of the two allotetraploids growing in proximity, and remain stable in a common garden. In addition, the sympatric populations essentially remain binary from the point of view of characteristic phenotypic traits, such as leaves and inflorescences—similar to ecological conditions (see also Paun et al., 2010, 2011).

Hence, the frequent gene flow does not appear to homogenize the phenotypes of the allopolyploids. This suggests that divergent ecological selection is active at particular loci, possibly some regulating gene expression such as *cis*‐acting promoters and *trans*‐acting transcription factors, and maintains the distinct ecological niches of the two sibling allopolyploids. Their distinct present ecophysiologies could be rooted in their independent origins involving the same diploid progenitor species, but different parental genotypes (Balao et al., 2017) and environmental settings, and might be maintained by stoichiometric requirements acting on complex regulatory pathways, which are known to be particularly strong for photosynthetic functions (Coate et al., 2012; Doyle & Coate, 2019). Furthermore, since the two allopolyploids formed at different times (Balao et al., 2016; Brandrud, 2019; Brandrud et al., 2020; Hawranek, 2021), they could be at different stages along the diploidization process. During the time since polyploidization, differences in ecology and geographic distribution might have led to differential genomic loss between the two allopolyploid species, resulting in incompatible sets of regulatory networks between the two sibling allopolyploids. Together or individually, these factors could explain how the strength of ecological segregation seems to be sufficient to maintain phenotypic divergence in the face of gene flow. This conclusion is also supported by the evidence that ecological differentiation is a major driver behind orchid diversification in general, independent of ploidy (e.g. Ackermann et al., 2007; Tupac Otero & Flanagan, 2006).

The pace of divergence of allopolyploids from their initial neopolyploid genomic background depends heavily on population parameters such as gene flow, generation time and mating systems (Orsucci et al., 2022), or on reproductive features such as the number of flowers per individual and the complexity of pollinator interactions. Given their widespread distribution, possibilities for long‐range dispersal due to their tiny endosperm‐less seeds, their fairly large effective population sizes [suggested to be in the range of thousands of individuals for either species by Hawranek (2021)] and the success of both polyploids, we assume that genetic drift stochastically shaping allele frequencies of these species is likely to have been a minor force for these established allopolyploids in the recent past and the present. However, around the time of their formations, when their populations were smaller, stochastic processes may have been more important. It remains unclear whether the ecophysiological differences between the allotetraploids are driven by selection on new polymorphisms, selection on standing variation from within the parental subgenomes, drift during early stages after allopolyploidization or a combination of these processes. Nevertheless, we suggest in this study that establishment of recurrent allopolyploids into distinct niches can happen relatively rapidly (te Beest et al., 2012) and this can significantly contribute to maintaining distinct phenotype and evolutionary trajectories, even when sharing the same ploidy, similar genetic backgrounds and in the face of a pervasive gene flow in sympatry.

## AUTHOR CONTRIBUTIONS

The study was designed by OP. Sampling and laboratory work was conducted by JB, TMW, FB, GB, WG, MH and OP. Bioinformatics and statistical analyses conducted by TMW and FB. Interpretation of the results was undertaken by TMW, FB, ET, GB, MH, WW, ARL and OP. The manuscript was drafted by TMW and OP, with first feedback from AL, and was revised and approved by all authors.

## FUNDING INFORMATION

This research was funded by the Austrian Science Fund (FWF) through the START grant Y661‐B16 to OP, and the doctoral programme (DK) grant W1225‐B20 to a faculty team including OP.

## CONFLICT OF INTEREST STATEMENT

The authors declare no conflict of interest.

### OPEN RESEARCH BADGES

This article has earned an Open Data badge for making publicly available the digitally‐shareable data necessary to reproduce the reported results. The data is available at URL https://www.ncbi.nlm.nih.gov/bioproject/PRJNA317244.

## BENEFIT SHARING STATEMENT

Benefits Generated: Benefits from this research have been shared as data and results on public databases as described above. Research collaborations were developed with scientists from the countries providing genetic samples, and all collaborators are included as co‐authors.

## Supporting information


Data S1:

## Data Availability

The raw Illumina sequencing data are deposited on NCBI SRA (GenBank BioProject PRJNA317244, accessions SRR17665593–SRR17665623). The new *D*. *incarnata* genome assembly and the underlying data are available from GenBank BioProject PRJNA934399. The table of counts and the bioinformatics scripts are available from GitHub (https://github.com/twolfe/dactylorhiza).
