# Supplementary material for: Recurrent allopolyploidizations diversify ecophysiological traits in marsh orchids (Dactylorhiza majalis s.l.)
Source: Mol Ecol. 2023 Jul 15;32(17):4777–90. doi: 10.1111/mec.17070 (PMC10947288; doi:10.1111/mec.17070)
Supplement: Supplementary file 1 — Data S1: [file MEC-32-4777-s001.pdf]

MOLECULAR ECOLOGY

Supplemental Information for:

Recurrent allopolyploidizations diversify eco-physiological traits in marsh orchids (*Dactylorhiza majalis* s.l.)

Thomas M. Wolfe, Francisco Balao, Emiliano Trucchi, Gert Bachmann, Wenjia Gu, Juliane Baar, Mikael Hedrén, Wolfram Weckwerth, Andrew R. Leitch, Ovidiu Paun

Table of Contents:

**Fig. S1**	Page 2
**Fig. S2**	Page 2
**Fig. S3**	Page 3
**Fig. S4**	Page 4
**Table S1**	Page 5
**Table S2**	Page 6
**Table S3**	Page 9
**Table S4**	Page 24

MOLECULAR ECOLOGY

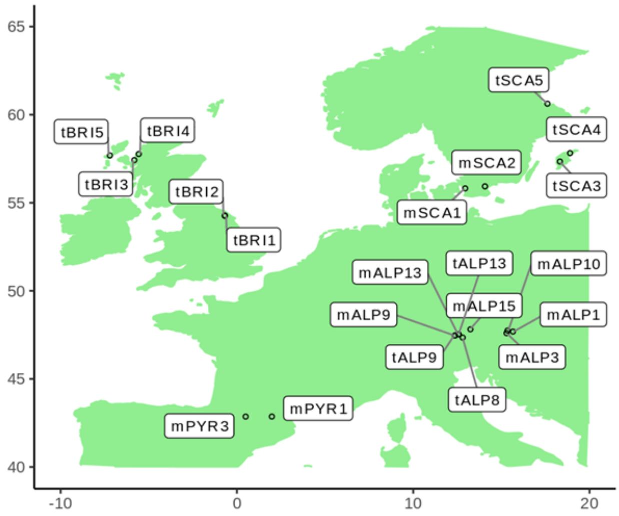

Figure S1. Populations sampled for the RNAseq experiment. The acronyms start with a species identifier: m, *D. majalis*; t, *D. traunsteineri*. There are six Alpine populations, four British populations, two populations in the Pyrenees and five Scandinavian populations.

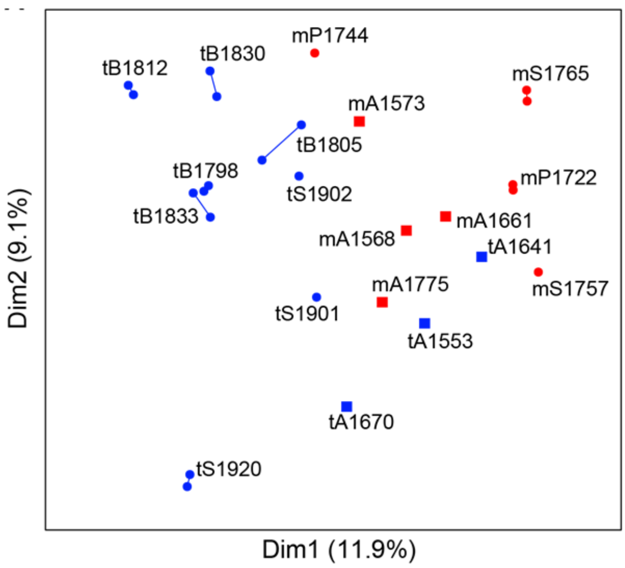

Figure S2. Principal component analysis (PCA) for gene expression variation for individuals of *D. majalis* (red symbols) and *D. traunsteineri* (blue) in a common garden setting. Expression data showes a closer overlap for individuals originating from the sympatric area in the Alps (squares; accession acronym contain “A”), compared with samples from other regions (filled circles; acronyms containing B - British Isles, S - Scandinavia, P - Pyrenees). Technical replicates are connected with lines.

MOLECULAR ECOLOGY

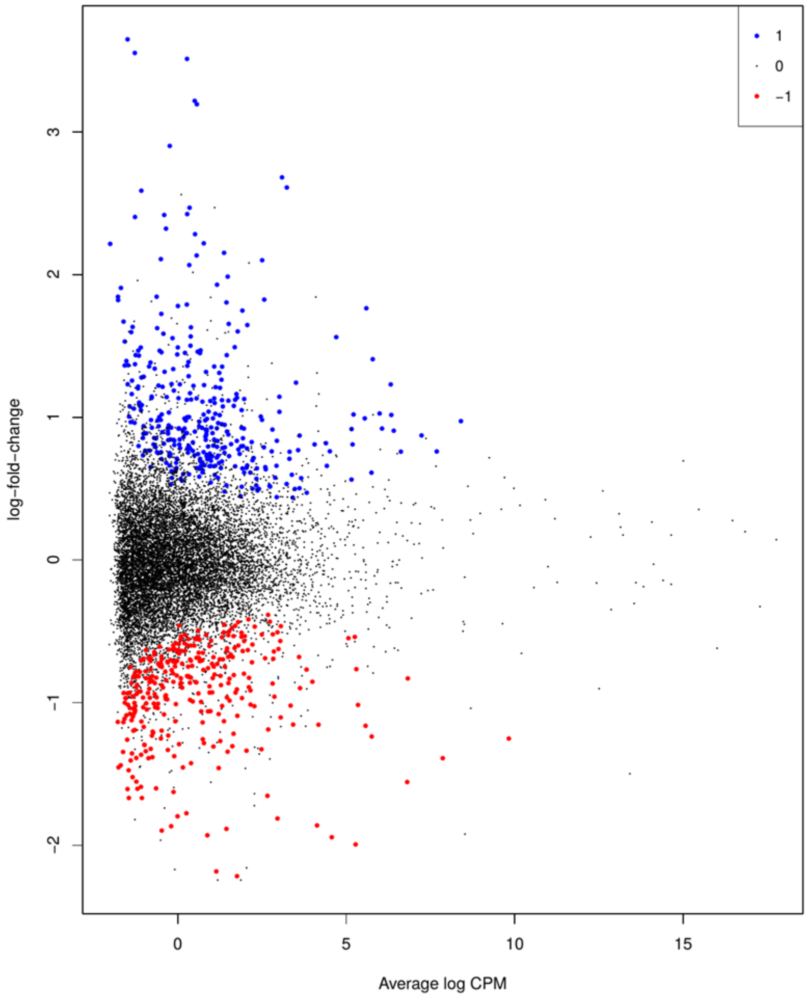

Figure S3. Visualization (i.e., MA plot) of the average counts per million on the x-axes and the log fold change on the y-axes (positive values = overexpressed in *D. traunsteineri*; negative values = overexpressed in *D. majalis*). Red points illustrate transcripts that are significantly more highly expressed in *D. majalis* and blue points illustrate transcripts that are more highly expressed in *D. traunsteineri*.

MOLECULAR ECOLOGY

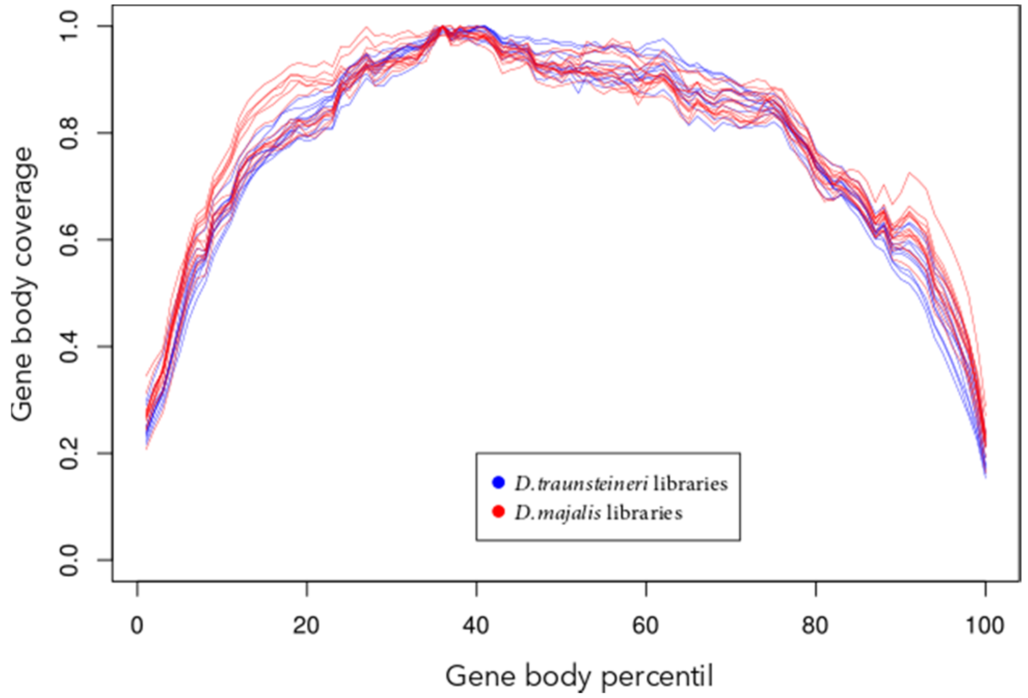

Figure S4. Transcriptome gene body coverage, showing that there are no 3' biases in coverage between our different samples. 5'-3' coverage biases are known to affect RNA-seq experiments and were estimated with *RSeQC*.

MOLECULAR ECOLOGY

Supplementary Tables

Table S1. The populations of *Dactylorhiza majalis* and *D. traunsteineri* we investigated here with different analyses. For leaf chemistry and RNA-seq, the individual accessions are indicated by acronyms including the species (m - *D. majalis*, t - *D. traunsteineri*), the region (A- the Alps, P - the Pyrenees, B - Britain, and S - Scandinavia), and the accession number. Note: the RNA-seq analyses have been performed in a common garden setup; here only the population of origin of the plants is indicated.

Species	Population ID	Country	Locality	Latitude	Longitude	Soil chemistry	Soil pH	Leaf chemistry	RNA-seq	Photosynthesis
majalis	mALP1	Austria	Altenberg	47.68553	15.6558	Yes	Yes	mA1031	mA1568	-
majalis	mALP3	Austria	Seewiesen	47.59644	15.29366	-	-	mA1083	-	-
majalis	mALP9	Austria	Kitzbühl	47.46155	12.3636	Yes	Yes	mA1651	-	Yes
majalis	mALP10	Austria	Mooshuben	47.74485	15.35082	Yes	Yes	mA1478	mA1573	-
majalis	mALP12	Austria	Wesenhof	47.31865	11.54238	-	Yes	-	-	-
majalis	mALP13	Austria	St Ulrich am Pillersee	47.53075	12.57522	Yes	Yes	mA1667	mA1661	Yes
majalis	mALP15	Austria	Fuschlsee	47.81454	13.24461	Yes	Yes	-	mA1775	-
majalis	mPYR1	France	D29, Belcaire to Espezel	42.8617	1.980923	Yes	Yes	mP1003, mP1004	mP1722	-
majalis	mPYR3	France	Bourg D'oueil	42.85863	0.495353	Yes	Yes	mP1744	mP1744	-
majalis	mSCA1	Sweden	Lanskrona, Saxtorp	55.8178	12.9456	Yes	Yes	mS1170	mS1765	-
majalis	mSCA2	Sweden	Kristianstadt, Lyngsjön	55.931	14.0683	Yes	Yes	mS1296	mS1757	-
traunsteineri	tALP8	Austria	Prilau	47.34304	12.80421	Yes	Yes	tA1384, tA1502	tA1553	Yes
traunsteineri	tALP9	Austria	Kitzbühl	47.46098	12.36565	Yes	Yes	tA1429, tA1431	tA1641	Yes
traunsteineri	tALP11	Austria	Gaisau	47.28165	11.18457	-	Yes	-	-	-
traunsteineri	tALP13	Austria	St Ulrich am Pillersee	47.52927	12.57853	Yes	Yes	tA1627	tA1670	Yes
traunsteineri	tBRI1	UK	Sand Dale	54.25277	-0.68508	Yes	Yes	tB1238, tB1239	tB1798	-
traunsteineri	tBRI2	UK	Seive Dale Fan	54.28157	-0.68977	-	Yes	tB1806	tB1805	-
traunsteineri	tBRI3	UK	Applecross	57.42202	-5.81932	Yes	Yes	tB1180	tB1812	-
traunsteineri	tBRI4	UK	Loch Kernsary	57.767	-5.569	-	Yes	tB1811	-	-
traunsteineri	tBRI5	UK	N Uist, Hebrides	57.6854	-7.20566	Yes	Yes	tB1829	tB1830, tB1833	-
traunsteineri	tSCA3	Sweden	Lojsthajd, Gotland	57.3402	18.32118	Yes	Yes	tS1400	tS1901, tS1902	-
traunsteineri	tSCA4	Sweden	Kauparve, Gotland	57.81707	18.89535	Yes	Yes	tS1416	-	-
traunsteineri	tSCA5	Sweden	Gårdskär, Uppland	60.62283	17.61094	Yes	Yes	-	tS1920	-

MOLECULAR ECOLOGY

Table S2. Results for permutation tests between measurements of various soil chemicals found in the soil for different populations across Europe. Permutation tests were performed because the distributions of the measured chemicals are unknown, we assume each measurement as being independent.

```
########## "N.NO3" ##########

        Exact Permutation Test (network algorithm)

data:  level by species
p-value = 1.149e-06
alternative hypothesis: true mean species=majalis - mean species=traunsteineri is not equal to 0
sample estimates:
mean species=majalis - mean species=traunsteineri
                                         32.65303

########## "N.NH4" ##########

        Permutation Test using Asymptotic Approximation

data:  level by species
Z = -0.37212, p-value = 0.7098
alternative hypothesis: true mean species=majalis - mean species=traunsteineri is not equal to 0
sample estimates:
mean species=majalis - mean species=traunsteineri
                                        -3.500559

########## "Al" ##########

        Permutation Test using Asymptotic Approximation

data:  level by species
Z = 2.212, p-value = 0.02697
alternative hypothesis: true mean species=majalis - mean species=traunsteineri is not equal to 0
sample estimates:
mean species=majalis - mean species=traunsteineri
                                         6.748555

########## "Ca" ##########

        Permutation Test using Asymptotic Approximation

data:  level by species
Z = -1.7507, p-value = 0.08
alternative hypothesis: true mean species=majalis - mean species=traunsteineri is not equal to 0
sample estimates:
mean species=majalis - mean species=traunsteineri
                                        -32.35937

########## "Cd" ##########

        Permutation Test using Asymptotic Approximation

data:  level by species
Z = 1.263, p-value = 0.2066
alternative hypothesis: true mean species=majalis - mean species=traunsteineri is not equal to 0
sample estimates:
mean species=majalis - mean species=traunsteineri
                                        0.1473309

########## "Cr" ##########

        Permutation Test using Asymptotic Approximation

data:  level by species
Z = 2.1042, p-value = 0.03536
alternative hypothesis: true mean species=majalis - mean species=traunsteineri is not equal to 0
sample estimates:
mean species=majalis - mean species=traunsteineri
                                         7.673745

########## "Cu" ##########

        Permutation Test using Asymptotic Approximation

data:  level by species
Z = -0.96389, p-value = 0.3351
alternative hypothesis: true mean species=majalis - mean species=traunsteineri is not equal to 0
sample estimates:
mean species=majalis - mean species=traunsteineri
                                        -16.20464
```

Table S2. Continued.

```
########## "Fe" ##########

        Permutation Test using Asymptotic Approximation

data:  level by species
Z = 1.1739, p-value = 0.2404
alternative hypothesis: true mean species=majalis - mean species=traunsteineri is not equal to 0
sample estimates:
mean species=majalis - mean species=traunsteineri
                                         5.935113

########## "K" ##########

        Permutation Test using Asymptotic Approximation

data:  level by species
Z = 3.1316, p-value = 0.001738
alternative hypothesis: true mean species=majalis - mean species=traunsteineri is not equal to 0
sample estimates:
mean species=majalis - mean species=traunsteineri
                                        0.7691066

########## "Mg" ##########

        Permutation Test using Asymptotic Approximation

data:  level by species
Z = -0.57622, p-value = 0.5645
alternative hypothesis: true mean species=majalis - mean species=traunsteineri is not equal to 0
sample estimates:
mean species=majalis - mean species=traunsteineri
                                        -3.772769

########## "Mn" ##########

        Permutation Test using Asymptotic Approximation

data:  level by species
Z = 0.57925, p-value = 0.5624
alternative hypothesis: true mean species=majalis - mean species=traunsteineri is not equal to 0
sample estimates:
mean species=majalis - mean species=traunsteineri
                                         138.7612

########## "Mo" ##########

        Permutation Test using Asymptotic Approximation

data:  level by species
Z = -0.73596, p-value = 0.4618
alternative hypothesis: true mean species=majalis - mean species=traunsteineri is not equal to 0
sample estimates:
mean species=majalis - mean species=traunsteineri
                                       -0.8096167

########## "Na" ##########

        Permutation Test using Asymptotic Approximation

data:  level by species
Z = -0.92668, p-value = 0.3541
alternative hypothesis: true mean species=majalis - mean species=traunsteineri is not equal to 0
sample estimates:
mean species=majalis - mean species=traunsteineri
                                        -55.72212

########## "Ni" ##########

        Permutation Test using Asymptotic Approximation

data:  level by species
Z = 2.212, p-value = 0.02696
alternative hypothesis: true mean species=majalis - mean species=traunsteineri is not equal to 0
sample estimates:
mean species=majalis - mean species=traunsteineri
                                         9.028801
```

MOLECULAR ECOLOGY

Table S2. Continued.

```
########## "P" ##########

        Permutation Test using Asymptotic Approximation

data:  level by species
Z = 3.4124, p-value = 0.000644
alternative hypothesis: true mean species=majalis - mean species=traunsteineri is not equal to 0
sample estimates:
mean species=majalis - mean species=traunsteineri
                                         416.8851

########## "Pb" ##########

        Permutation Test using Asymptotic Approximation

data:  level by species
Z = 0.7782, p-value = 0.4365

alternative hypothesis: true mean species=majalis - mean species=traunsteineri is not equal to 0
sample estimates:
```

MOLECULAR ECOLOGY

Table S3. Table showing enrichment GO terms in Biological Process with their corresponding name. The fourth column is the number of significant transcripts differentially expressed between *D. traunsteineri* and *D. majalis*; the fifth column is the adjusted p-value for the corresponding GO enrichment, the seventh column is corresponding z-score. The last two columns include the gene names and their corresponding log fold change.

category	ID	GO-term	count	adj_pval	zscore	genes	logFC
BP	GO:1903508	positive regulation of nucleic acid-temp...	8	0.00011	-2.272629253	Dinc061141-RA	-0.8132843937
BP	GO:1903508	positive regulation of nucleic acid-temp...	8	0.00011	-2.272629253	Dinc080279-RA	-0.5540832048
BP	GO:1903508	positive regulation of nucleic acid-temp...	8	0.00011	-2.272629253	Dinc082500-RA	-1.590062266
BP	GO:1903508	positive regulation of nucleic acid-temp...	8	0.00011	-2.272629253	Dinc082807-RA	-1.18770199
BP	GO:1903508	positive regulation of nucleic acid-temp...	8	0.00011	-2.272629253	Dinc086527-RB	1.456173629
BP	GO:1903508	positive regulation of nucleic acid-temp...	8	0.00011	-2.272629253	Dinc086672-RA	-1.865922633
BP	GO:1903508	positive regulation of nucleic acid-temp...	8	0.00011	-2.272629253	Dinc108568-RA	-1.092253692
BP	GO:1903508	positive regulation of nucleic acid-temp...	8	0.00011	-2.272629253	Dinc133462-RA	-0.7808316746
BP	GO:0055114	oxidation-reduction process	72	0.00011	1.980153565	Dinc061341-RA	0.4373025248
BP	GO:0055114	oxidation-reduction process	72	0.00011	1.980153565	Dinc062201-RA	1.123356389
BP	GO:0055114	oxidation-reduction process	72	0.00011	1.980153565	Dinc064072-RA	-0.9355098938
BP	GO:0055114	oxidation-reduction process	72	0.00011	1.980153565	Dinc065981-RA	0.789801727
BP	GO:0055114	oxidation-reduction process	72	0.00011	1.980153565	Dinc067566-RA	0.7412605539
BP	GO:0055114	oxidation-reduction process	72	0.00011	1.980153565	Dinc069233-RA	1.633648099
BP	GO:0055114	oxidation-reduction process	72	0.00011	1.980153565	Dinc069517-RA	0.6598007837
BP	GO:0055114	oxidation-reduction process	72	0.00011	1.980153565	Dinc071434-RA	0.7593059306
BP	GO:0055114	oxidation-reduction process	72	0.00011	1.980153565	Dinc072469-RA	1.467198545
BP	GO:0055114	oxidation-reduction process	72	0.00011	1.980153565	Dinc073372-RA	-1.296952805
BP	GO:0055114	oxidation-reduction process	72	0.00011	1.980153565	Dinc074834-RA	2.101264452
BP	GO:0055114	oxidation-reduction process	72	0.00011	1.980153565	Dinc074862-RA	0.8800621915

MOLECULAR ECOLOGY

BP	GO:0055114	oxidation-reduction process	72	0.00011	1.980153565	Dinc074964-RA	-0.4938499332
BP	GO:0055114	oxidation-reduction process	72	0.00011	1.980153565	Dinc076296-RA	-0.7169375149
BP	GO:0055114	oxidation-reduction process	72	0.00011	1.980153565	Dinc077209-RA	3.512629011
BP	GO:0055114	oxidation-reduction process	72	0.00011	1.980153565	Dinc084360-RA	0.7613860239
BP	GO:0055114	oxidation-reduction process	72	0.00011	1.980153565	Dinc084769-RA	0.7566842943
BP	GO:0055114	oxidation-reduction process	72	0.00011	1.980153565	Dinc085390-RA	0.7985370953
BP	GO:0055114	oxidation-reduction process	72	0.00011	1.980153565	Dinc090102-RA	1.0802929
BP	GO:0055114	oxidation-reduction process	72	0.00011	1.980153565	Dinc091072-RA	0.7439200922
BP	GO:0055114	oxidation-reduction process	72	0.00011	1.980153565	Dinc093427-RA	0.9813850451
BP	GO:0055114	oxidation-reduction process	72	0.00011	1.980153565	Dinc093958-RA	0.6122401898
BP	GO:0055114	oxidation-reduction process	72	0.00011	1.980153565	Dinc095511-RA	1.206590806
BP	GO:0055114	oxidation-reduction process	72	0.00011	1.980153565	Dinc097557-RA	-0.6261377154
BP	GO:0055114	oxidation-reduction process	72	0.00011	1.980153565	Dinc098057-RA	0.6280244623
BP	GO:0055114	oxidation-reduction process	72	0.00011	1.980153565	Dinc098792-RA	-0.6373337471
BP	GO:0055114	oxidation-reduction process	72	0.00011	1.980153565	Dinc100785-RA	-1.9430961
BP	GO:0055114	oxidation-reduction process	72	0.00011	1.980153565	Dinc101783-RA	-1.775329891
BP	GO:0055114	oxidation-reduction process	72	0.00011	1.980153565	Dinc102471-RA	0.5203470562
BP	GO:0055114	oxidation-reduction process	72	0.00011	1.980153565	Dinc106327-RA	-0.5925328326
BP	GO:0055114	oxidation-reduction process	72	0.00011	1.980153565	Dinc108385-RA	0.613040176
BP	GO:0055114	oxidation-reduction process	72	0.00011	1.980153565	Dinc108497-RA	-0.6311270517
BP	GO:0055114	oxidation-reduction process	72	0.00011	1.980153565	Dinc108753-RA	0.7066373898
BP	GO:0055114	oxidation-reduction process	72	0.00011	1.980153565	Dinc109223-RA	-0.5586103738
BP	GO:0055114	oxidation-reduction process	72	0.00011	1.980153565	Dinc111345-RA	1.233519339
BP	GO:0055114	oxidation-reduction process	72	0.00011	1.980153565	Dinc111364-RA	0.9733462292
BP	GO:0055114	oxidation-reduction process	72	0.00011	1.980153565	Dinc111643-RA	-1.196256011

BP	GO:0055114	oxidation-reduction process	72	0.00011	1.980153565	Dinc114258-RA	0.8267448131
BP	GO:0055114	oxidation-reduction process	72	0.00011	1.980153565	Dinc114607-RA	0.522588174
BP	GO:0055114	oxidation-reduction process	72	0.00011	1.980153565	Dinc115982-RB	-1.307455953
BP	GO:0055114	oxidation-reduction process	72	0.00011	1.980153565	Dinc115983-RA	-1.250909075
BP	GO:0055114	oxidation-reduction process	72	0.00011	1.980153565	Dinc116779-RA	-1.34370264
BP	GO:0055114	oxidation-reduction process	72	0.00011	1.980153565	Dinc119051-RA	-0.8121168586
BP	GO:0055114	oxidation-reduction process	72	0.00011	1.980153565	Dinc119782-RA	1.128737705
BP	GO:0055114	oxidation-reduction process	72	0.00011	1.980153565	Dinc121039-RB	2.682213066
BP	GO:0055114	oxidation-reduction process	72	0.00011	1.980153565	Dinc122110-RA	-0.7840035927
BP	GO:0055114	oxidation-reduction process	72	0.00011	1.980153565	Dinc123050-RA	0.7282238924
BP	GO:0055114	oxidation-reduction process	72	0.00011	1.980153565	Dinc123601-RA	-0.7578578865
BP	GO:0055114	oxidation-reduction process	72	0.00011	1.980153565	Dinc12376-RA	-0.7852699473
BP	GO:0055114	oxidation-reduction process	72	0.00011	1.980153565	Dinc125815-RA	-0.7647729863
BP	GO:0055114	oxidation-reduction process	72	0.00011	1.980153565	Dinc126588-RA	0.7333330285
BP	GO:0055114	oxidation-reduction process	72	0.00011	1.980153565	Dinc126954-RA	1.132482781
BP	GO:0055114	oxidation-reduction process	72	0.00011	1.980153565	Dinc127388-RA	1.562324179
BP	GO:0055114	oxidation-reduction process	72	0.00011	1.980153565	Dinc129156-RA	0.8620306135
BP	GO:0055114	oxidation-reduction process	72	0.00011	1.980153565	Dinc129215-RA	0.7608185282
BP	GO:0055114	oxidation-reduction process	72	0.00011	1.980153565	Dinc129858-RA	0.4996691155
BP	GO:0055114	oxidation-reduction process	72	0.00011	1.980153565	Dinc129949-RA	0.9817420776
BP	GO:0055114	oxidation-reduction process	72	0.00011	1.980153565	Dinc131192-RA	1.790649219
BP	GO:0055114	oxidation-reduction process	72	0.00011	1.980153565	Dinc134486-RA	0.9699568561
BP	GO:0055114	oxidation-reduction process	72	0.00011	1.980153565	Dinc134844-RA	-1.01341594
BP	GO:0055114	oxidation-reduction process	72	0.00011	1.980153565	Dinc136090-RA	1.050426387
BP	GO:0055114	oxidation-reduction process	72	0.00011	1.980153565	Dinc136843-RA	-1.8845327

BP	GO:0055114	oxidation-reduction process	72	0.00011	1.980153565	Dinc138027-RA	0.8350407383
BP	GO:0055114	oxidation-reduction process	72	0.00011	1.980153565	Dinc138415-RA	-0.7419921538
BP	GO:0055114	oxidation-reduction process	72	0.00011	1.980153565	Dinc27047-RA	-1.037987044
BP	GO:0055114	oxidation-reduction process	72	0.00011	1.980153565	Dinc34785-RA	0.9789331816
BP	GO:0055114	oxidation-reduction process	72	0.00011	1.980153565	Dinc36662-RA	-0.8770024055
BP	GO:0055114	oxidation-reduction process	72	0.00011	1.980153565	Dinc42136-RA	-0.6922598207
BP	GO:0055114	oxidation-reduction process	72	0.00011	1.980153565	Dinc46235-RA	-1.210577412
BP	GO:0055114	oxidation-reduction process	72	0.00011	1.980153565	Dinc48160-RA	-0.6796090537
BP	GO:0055114	oxidation-reduction process	72	0.00011	1.980153565	Dinc53263-RA	1.211823864
BP	GO:0055114	oxidation-reduction process	72	0.00011	1.980153565	Dinc54703-RA	-0.8300200202
BP	GO:0015995	chlorophyll biosynthetic process	5	0.00019	1.922160893	Dinc093427-RA	0.9813850451
BP	GO:0015995	chlorophyll biosynthetic process	5	0.00019	1.922160893	Dinc093958-RA	0.6122401898
BP	GO:0015995	chlorophyll biosynthetic process	5	0.00019	1.922160893	Dinc127388-RA	1.562324179
BP	GO:0015995	chlorophyll biosynthetic process	5	0.00019	1.922160893	Dinc129858-RA	0.4996691155
BP	GO:0015995	chlorophyll biosynthetic process	5	0.00019	1.922160893	Dinc131805-RA	0.6424638913
BP	GO:0006809	nitric oxide biosynthetic process	2	0.00021	-1.80903726	Dinc115982-RB	-1.307455953
BP	GO:0006809	nitric oxide biosynthetic process	2	0.00021	-1.80903726	Dinc115983-RA	-1.250909075
BP	GO:0018298	protein-chromophore linkage	7	0.00022	2.918112538	Dinc063551-RA	1.016712205
BP	GO:0018298	protein-chromophore linkage	7	0.00022	2.918112538	Dinc082292-RA	0.9215198616
BP	GO:0018298	protein-chromophore linkage	7	0.00022	2.918112538	Dinc085612-RA	0.9926283539
BP	GO:0018298	protein-chromophore linkage	7	0.00022	2.918112538	Dinc110117-RA	1.646926195
BP	GO:0018298	protein-chromophore linkage	7	0.00022	2.918112538	Dinc118447-RA	0.8732122273
BP	GO:0018298	protein-chromophore linkage	7	0.00022	2.918112538	Dinc129156-RA	0.8620306135
BP	GO:0018298	protein-chromophore linkage	7	0.00022	2.918112538	Dinc39686-RA	1.407570617
BP	GO:0009416	response to light stimulus	14	0.00025	2.625787606	Dinc063551-RA	1.016712205

BP	GO:0009416	response to light stimulus	14	0.00025	2.625787606	Dinc066269-RA	0.6508601416
BP	GO:0009416	response to light stimulus	14	0.00025	2.625787606	Dinc069233-RA	1.633648099
BP	GO:0009416	response to light stimulus	14	0.00025	2.625787606	Dinc074862-RA	0.8800621915
BP	GO:0009416	response to light stimulus	14	0.00025	2.625787606	Dinc078468-RA	-0.6073328114
BP	GO:0009416	response to light stimulus	14	0.00025	2.625787606	Dinc082292-RA	0.9215198616
BP	GO:0009416	response to light stimulus	14	0.00025	2.625787606	Dinc085612-RA	0.9926283539
BP	GO:0009416	response to light stimulus	14	0.00025	2.625787606	Dinc088481-RA	-0.8025693012
BP	GO:0009416	response to light stimulus	14	0.00025	2.625787606	Dinc095654-RA	0.8186680859
BP	GO:0009416	response to light stimulus	14	0.00025	2.625787606	Dinc110117-RA	1.646926195
BP	GO:0009416	response to light stimulus	14	0.00025	2.625787606	Dinc118447-RA	0.8732122273
BP	GO:0009416	response to light stimulus	14	0.00025	2.625787606	Dinc128726-RA	1.36102387
BP	GO:0009416	response to light stimulus	14	0.00025	2.625787606	Dinc131512-RA	-0.9681321423
BP	GO:0009416	response to light stimulus	14	0.00025	2.625787606	Dinc39686-RA	1.407570617
BP	GO:0015979	photosynthesis	24	0.00052	3.802307751	Dinc062437-RA	0.9292113613
BP	GO:0015979	photosynthesis	24	0.00052	3.802307751	Dinc063551-RA	1.016712205
BP	GO:0015979	photosynthesis	24	0.00052	3.802307751	Dinc069517-RA	0.6598007837
BP	GO:0015979	photosynthesis	24	0.00052	3.802307751	Dinc071434-RA	0.7593059306
BP	GO:0015979	photosynthesis	24	0.00052	3.802307751	Dinc082292-RA	0.9215198616
BP	GO:0015979	photosynthesis	24	0.00052	3.802307751	Dinc085612-RA	0.9926283539
BP	GO:0015979	photosynthesis	24	0.00052	3.802307751	Dinc093427-RA	0.9813850451
BP	GO:0015979	photosynthesis	24	0.00052	3.802307751	Dinc093958-RA	0.6122401898
BP	GO:0015979	photosynthesis	24	0.00052	3.802307751	Dinc097505-RA	1.019985448
BP	GO:0015979	photosynthesis	24	0.00052	3.802307751	Dinc108420-RA	1.038450435
BP	GO:0015979	photosynthesis	24	0.00052	3.802307751	Dinc110117-RA	1.646926195
BP	GO:0015979	photosynthesis	24	0.00052	3.802307751	Dinc111364-RA	0.9733462292

MOLECULAR ECOLOGY

BP	GO:0015979	photosynthesis	24	0.00052	3.802307751	Dinc118447-RA	0.8732122273
BP	GO:0015979	photosynthesis	24	0.00052	3.802307751	Dinc123050-RA	0.7282238924
BP	GO:0015979	photosynthesis	24	0.00052	3.802307751	Dinc125308-RA	0.9179463095
BP	GO:0015979	photosynthesis	24	0.00052	3.802307751	Dinc127388-RA	1.562324179
BP	GO:0015979	photosynthesis	24	0.00052	3.802307751	Dinc129156-RA	0.8620306135
BP	GO:0015979	photosynthesis	24	0.00052	3.802307751	Dinc129215-RA	0.7608185282
BP	GO:0015979	photosynthesis	24	0.00052	3.802307751	Dinc131805-RA	0.6424638913
BP	GO:0015979	photosynthesis	24	0.00052	3.802307751	Dinc136090-RA	1.050426387
BP	GO:0015979	photosynthesis	24	0.00052	3.802307751	Dinc136843-RA	-1.8845327
BP	GO:0015979	photosynthesis	24	0.00052	3.802307751	Dinc138027-RA	0.8350407383
BP	GO:0015979	photosynthesis	24	0.00052	3.802307751	Dinc39686-RA	1.407570617
BP	GO:0015979	photosynthesis	24	0.00052	3.802307751	Dinc48160-RA	-0.6796090537
BP	GO:0015996	chlorophyll catabolic process	3	8.00E-04	-1.356227854	Dinc074964-RA	-0.4938499332
BP	GO:0015996	chlorophyll catabolic process	3	8.00E-04	-1.356227854	Dinc082582-RA	-1.21787187
BP	GO:0015996	chlorophyll catabolic process	3	8.00E-04	-1.356227854	Dinc098792-RA	-0.6373337471
BP	GO:0051938	L-glutamate import	2	0.00126	-1.732576076	Dinc092050-RA	-1.458240797
BP	GO:0051938	L-glutamate import	2	0.00126	-1.732576076	Dinc20072-RA	-0.9919917871
BP	GO:0042128	nitrate assimilation	2	0.00126	-1.80903726	Dinc115982-RB	-1.307455953
BP	GO:0042128	nitrate assimilation	2	0.00126	-1.80903726	Dinc115983-RA	-1.250909075
BP	GO:0080167	response to karrikin	3	0.00295	1.846846708	Dinc069319-RA	1.821945965
BP	GO:0080167	response to karrikin	3	0.00295	1.846846708	Dinc112983-RA	0.717533642
BP	GO:0080167	response to karrikin	3	0.00295	1.846846708	Dinc117592-RA	0.6593527247
BP	GO:1903401	L-lysine transmembrane transport	2	0.00427	-1.732576076	Dinc092050-RA	-1.458240797
BP	GO:1903401	L-lysine transmembrane transport	2	0.00427	-1.732576076	Dinc20072-RA	-0.9919917871
BP	GO:0015813	L-glutamate transmembrane transport	2	0.00427	-1.732576076	Dinc092050-RA	-1.458240797

BP	GO:0015813	L-glutamate transmembrane transport	2	0.00427	-1.732576076	Dinc20072-RA	-0.9919917871
BP	GO:0045490	pectin catabolic process	5	0.00641	2.891391467	Dinc060343-RA	3.554188831
BP	GO:0045490	pectin catabolic process	5	0.00641	2.891391467	Dinc072575-RA	2.611061858
BP	GO:0045490	pectin catabolic process	5	0.00641	2.891391467	Dinc082995-RA	-1.89668597
BP	GO:0045490	pectin catabolic process	5	0.00641	2.891391467	Dinc10092-RA	1.076256962
BP	GO:0045490	pectin catabolic process	5	0.00641	2.891391467	Dinc29778-RA	1.120526189
BP	GO:0009408	response to heat	6	0.01349	-2.341390071	Dinc072616-RA	-0.8158515417
BP	GO:0009408	response to heat	6	0.01349	-2.341390071	Dinc074862-RA	0.8800621915
BP	GO:0009408	response to heat	6	0.01349	-2.341390071	Dinc082807-RA	-1.18770199
BP	GO:0009408	response to heat	6	0.01349	-2.341390071	Dinc083258-RA	-1.336562116
BP	GO:0009408	response to heat	6	0.01349	-2.341390071	Dinc092213-RA	-2.182903815
BP	GO:0009408	response to heat	6	0.01349	-2.341390071	Dinc108568-RA	-1.092253692
BP	GO:0019305	dTDP-rhamnose biosynthetic process	1	0.01463	0.5203470562	Dinc102471-RA	0.5203470562
BP	GO:0035066	positive regulation of histone acetylati...	1	0.01463	-0.5540832048	Dinc080279-RA	-0.5540832048
BP	GO:0000301	retrograde transport, vesicle recycling ...	1	0.01463	-0.6234698271	Dinc119789-RA	-0.6234698271
BP	GO:0046167	glycerol-3-phosphate biosynthetic proces...	1	0.01463	0.6593527247	Dinc117592-RA	0.6593527247
BP	GO:0019447	D-cysteine catabolic process	1	0.01463	0.6639482574	Dinc061226-RA	0.6639482574
BP	GO:0015720	allantoin transport	1	0.01463	-0.7477888692	Dinc079735-RB	-0.7477888692
BP	GO:0034051	negative regulation of plant-type hypers...	1	0.01463	0.9218534849	Dinc41235-RA	0.9218534849
BP	GO:1903791	uracil transmembrane transport	1	0.01463	-0.7477888692	Dinc079735-RB	-0.7477888692
BP	GO:0046322	negative regulation of fatty acid oxidat...	1	0.01463	0.8800621915	Dinc074862-RA	0.8800621915
BP	GO:0051391	tRNA acetylation	1	0.01463	-1.454000623	Dinc091776-RA	-1.454000623
BP	GO:0070914	UV-damage excision repair	1	0.01463	-0.9681321423	Dinc131512-RA	-0.9681321423
BP	GO:0010253	UDP-rhamnose biosynthetic process	1	0.01463	0.5203470562	Dinc102471-RA	0.5203470562
BP	GO:0010213	non-photoreactive DNA repair	1	0.01463	-0.9681321423	Dinc131512-RA	-0.9681321423

MOLECULAR ECOLOGY

BP	GO:0010230	alternative respiration	1	0.01463	-0.6922598207	Dinc42136-RA	-0.6922598207
BP	GO:0010315	auxin efflux	2	0.01497	1.015271734	Dinc066269-RA	0.6508601416
BP	GO:0010315	auxin efflux	2	0.01497	1.015271734	Dinc34122-RA	0.7849509135
BP	GO:0009773	photosynthetic electron transport in pho...	2	0.02238	1.20931323	Dinc069517-RA	0.6598007837
BP	GO:0009773	photosynthetic electron transport in pho...	2	0.02238	1.20931323	Dinc136090-RA	1.050426387
BP	GO:0018108	peptidyl-tyrosine phosphorylation	5	0.02459	-0.06268894031	Dinc082786-RA	1.116736985
BP	GO:0018108	peptidyl-tyrosine phosphorylation	5	0.02459	-0.06268894031	Dinc089930-RA	1.489447846
BP	GO:0018108	peptidyl-tyrosine phosphorylation	5	0.02459	-0.06268894031	Dinc095889-RA	-1.365125995
BP	GO:0018108	peptidyl-tyrosine phosphorylation	5	0.02459	-0.06268894031	Dinc103893-RA	-0.6953951752
BP	GO:0018108	peptidyl-tyrosine phosphorylation	5	0.02459	-0.06268894031	Dinc47062-RA	-0.6858403922
BP	GO:0006122	mitochondrial electron transport, ubiqui...	2	0.02799	-0.06836802747	Dinc126588-RA	0.7333330285
BP	GO:0006122	mitochondrial electron transport, ubiqui...	2	0.02799	-0.06836802747	Dinc54703-RA	-0.8300200202
BP	GO:0010431	seed maturation	2	0.02899	0.09351591836	Dinc074862-RA	0.8800621915
BP	GO:0010431	seed maturation	2	0.02899	0.09351591836	Dinc125403-RA	-0.7478107115
BP	GO:0045017	glycerolipid biosynthetic process	4	0.02904	-0.1238387045	Dinc114293-RA	-0.5678854657
BP	GO:0045017	glycerolipid biosynthetic process	4	0.02904	-0.1238387045	Dinc114296-RA	-1.130635452
BP	GO:0045017	glycerolipid biosynthetic process	4	0.02904	-0.1238387045	Dinc130308-RA	-0.9740615265
BP	GO:0045017	glycerolipid biosynthetic process	4	0.02904	-0.1238387045	Dinc131802-RA	2.424905035
BP	GO:0009693	ethylene biosynthetic process	1	0.02905	0.6639482574	Dinc061226-RA	0.6639482574
BP	GO:0015741	fumarate transport	1	0.02905	0.8966019239	Dinc101621-RA	0.8966019239
BP	GO:1904812	rRNA acetylation involved in maturation ...	1	0.02905	-1.454000623	Dinc091776-RA	-1.454000623
BP	GO:0051176	positive regulation of sulfur metabolic ...	1	0.02905	-0.7419921538	Dinc138415-RA	-0.7419921538
BP	GO:1901562	response to paraquat	1	0.02905	0.8800621915	Dinc074862-RA	0.8800621915
BP	GO:0045900	negative regulation of translational elo...	1	0.02905	0.7817877427	Dinc130369-RA	0.7817877427
BP	GO:0036258	multivesicular body assembly	1	0.02905	-0.5653950227	Dinc098675-RA	-0.5653950227

MOLECULAR ECOLOGY

BP	GO:0042542	response to hydrogen peroxide	2	0.03099	-2.488638226	Dinc083258-RA	-1.336562116
BP	GO:0042542	response to hydrogen peroxide	2	0.03099	-2.488638226	Dinc092213-RA	-2.182903815
BP	GO:0010207	photosystem II assembly	2	0.03099	1.272275285	Dinc108420-RA	1.038450435
BP	GO:0010207	photosystem II assembly	2	0.03099	1.272275285	Dinc129215-RA	0.7608185282
BP	GO:0046854	phosphatidylinositol phosphorylation	3	0.03311	-0.2494418903	Dinc074902-RA	0.927299539
BP	GO:0046854	phosphatidylinositol phosphorylation	3	0.03311	-0.2494418903	Dinc098675-RA	-0.5653950227
BP	GO:0046854	phosphatidylinositol phosphorylation	3	0.03311	-0.2494418903	Dinc100169-RA	-0.7939505437
BP	GO:0006662	glycerol ether metabolic process	2	0.04068	0.9756531397	Dinc091072-RA	0.7439200922
BP	GO:0006662	glycerol ether metabolic process	2	0.04068	0.9756531397	Dinc137274-RA	0.6358618101
BP	GO:0071555	cell wall organization	10	0.04129	3.731743143	Dinc062437-RA	0.9292113613
BP	GO:0071555	cell wall organization	10	0.04129	3.731743143	Dinc063696-RA	1.825279683
BP	GO:0071555	cell wall organization	10	0.04129	3.731743143	Dinc069443-RA	2.418343673
BP	GO:0071555	cell wall organization	10	0.04129	3.731743143	Dinc082995-RA	-1.89668597
BP	GO:0071555	cell wall organization	10	0.04129	3.731743143	Dinc083646-RA	0.7897796325
BP	GO:0071555	cell wall organization	10	0.04129	3.731743143	Dinc108385-RA	0.613040176
BP	GO:0071555	cell wall organization	10	0.04129	3.731743143	Dinc122161-RA	1.986122829
BP	GO:0071555	cell wall organization	10	0.04129	3.731743143	Dinc29778-RA	1.120526189
BP	GO:0071555	cell wall organization	10	0.04129	3.731743143	Dinc38121-RA	0.7968090533
BP	GO:0071555	cell wall organization	10	0.04129	3.731743143	Dinc41693-RA	3.218381347
BP	GO:0090691	formation of plant organ boundary	1	0.04326	0.6508601416	Dinc066269-RA	0.6508601416
BP	GO:0019805	quinolinate biosynthetic process	1	0.04326	-0.7419921538	Dinc138415-RA	-0.7419921538
BP	GO:0070676	intralumenal vesicle formation	1	0.04326	-0.5653950227	Dinc098675-RA	-0.5653950227
BP	GO:0009294	DNA mediated transformation	1	0.04326	-0.6563208407	Dinc126831-RA	-0.6563208407
BP	GO:0015742	alpha-ketoglutarate transport	1	0.04326	0.4987257115	Dinc118144-RA	0.4987257115
BP	GO:0006788	heme oxidation	1	0.04326	0.9699568561	Dinc134486-RA	0.9699568561

BP	GO:0071422	succinate transmembrane transport	1	0.04326	0.8966019239	Dinc101621-RA	0.8966019239
BP	GO:0010540	basipetal auxin transport	1	0.04326	0.6508601416	Dinc066269-RA	0.6508601416
BP	GO:0010541	acropetal auxin transport	1	0.04326	0.6508601416	Dinc066269-RA	0.6508601416
BP	GO:0090391	granum assembly	1	0.04326	0.717533642	Dinc112983-RA	0.717533642
BP	GO:0031640	killing of cells of other organism	1	0.04326	1.001190761	Dinc110851-RA	1.001190761
BP	GO:0010019	chloroplast-nucleus signaling pathway	1	0.04326	0.9145027434	Dinc128770-RA	0.9145027434
BP	GO:0010188	response to microbial phytotoxin	1	0.04326	0.6593527247	Dinc117592-RA	0.6593527247
BP	GO:0043481	anthocyanin accumulation in tissues in r...	1	0.04326	0.6508601416	Dinc066269-RA	0.6508601416
BP	GO:0010117	photoprotection	1	0.04326	0.8800621915	Dinc074862-RA	0.8800621915
BP	GO:0009807	lignan biosynthetic process	1	0.04326	1.206590806	Dinc095511-RA	1.206590806
BP	GO:0019323	pentose catabolic process	1	0.04326	0.4406984246	Dinc119830-RA	0.4406984246
BP	GO:0006294	nucleotide-excision repair, preincision ...	1	0.04326	-0.9681321423	Dinc131512-RA	-0.9681321423
BP	GO:0006296	nucleotide-excision repair, DNA incision...	1	0.04326	-0.9681321423	Dinc131512-RA	-0.9681321423
BP	GO:0022904	respiratory electron transport chain	7	0.04343	-1.644838635	Dinc064072-RA	-0.9355098938
BP	GO:0022904	respiratory electron transport chain	7	0.04343	-1.644838635	Dinc073372-RA	-1.296952805
BP	GO:0022904	respiratory electron transport chain	7	0.04343	-1.644838635	Dinc076296-RA	-0.7169375149
BP	GO:0022904	respiratory electron transport chain	7	0.04343	-1.644838635	Dinc097557-RA	-0.6261377154
BP	GO:0022904	respiratory electron transport chain	7	0.04343	-1.644838635	Dinc126588-RA	0.7333330285
BP	GO:0022904	respiratory electron transport chain	7	0.04343	-1.644838635	Dinc48160-RA	-0.6796090537
BP	GO:0022904	respiratory electron transport chain	7	0.04343	-1.644838635	Dinc54703-RA	-0.8300200202
BP	GO:0009772	photosynthetic electron transport in pho...	3	0.04665	0.4862572967	Dinc069517-RA	0.6598007837
BP	GO:0009772	photosynthetic electron transport in pho...	3	0.04665	0.4862572967	Dinc129156-RA	0.8620306135
BP	GO:0009772	photosynthetic electron transport in pho...	3	0.04665	0.4862572967	Dinc48160-RA	-0.6796090537
BP	GO:0033499	galactose catabolic process via UDP-gala...	2	0.04769	1.704980564	Dinc088028-RA	1.122693445
BP	GO:0033499	galactose catabolic process via UDP-gala...	2	0.04769	1.704980564	Dinc103654-RA	1.288513193

BP	GO:0009409	response to cold	4	0.04779	1.490577148	Dinc069517-RA	0.6598007837
BP	GO:0009409	response to cold	4	0.04779	1.490577148	Dinc095654-RA	0.8186680859
BP	GO:0009409	response to cold	4	0.04779	1.490577148	Dinc111683-RA	0.7274285085
BP	GO:0009409	response to cold	4	0.04779	1.490577148	Dinc133819-RA	0.7752569184
BP	GO:0009060	aerobic respiration	7	0.05052	-1.549202622	Dinc073372-RA	-1.296952805
BP	GO:0009060	aerobic respiration	7	0.05052	-1.549202622	Dinc076296-RA	-0.7169375149
BP	GO:0009060	aerobic respiration	7	0.05052	-1.549202622	Dinc097557-RA	-0.6261377154
BP	GO:0009060	aerobic respiration	7	0.05052	-1.549202622	Dinc123601-RA	-0.7578578865
BP	GO:0009060	aerobic respiration	7	0.05052	-1.549202622	Dinc126588-RA	0.7333330285
BP	GO:0009060	aerobic respiration	7	0.05052	-1.549202622	Dinc138415-RA	-0.7419921538
BP	GO:0009060	aerobic respiration	7	0.05052	-1.549202622	Dinc42136-RA	-0.6922598207
BP	GO:0043085	positive regulation of catalytic activit...	4	0.0518	0.2998199972	Dinc091072-RA	0.7439200922
BP	GO:0043085	positive regulation of catalytic activit...	4	0.0518	0.2998199972	Dinc123454-RA	-0.5060436796
BP	GO:0043085	positive regulation of catalytic activit...	4	0.0518	0.2998199972	Dinc128985-RA	1.103755736
BP	GO:0043085	positive regulation of catalytic activit...	4	0.0518	0.2998199972	Dinc138415-RA	-0.7419921538
BP	GO:0070413	trehalose metabolism in response to stre...	2	0.0551	1.81096869	Dinc069563-RA	1.338389981
BP	GO:0070413	trehalose metabolism in response to stre...	2	0.0551	1.81096869	Dinc105668-RA	1.222706502
BP	GO:0010731	protein glutathionylation	1	0.05726	-0.8262182246	Dinc078007-RA	-0.8262182246
BP	GO:0009269	response to desiccation	1	0.05726	0.6461679457	Dinc128254-RA	0.6461679457
BP	GO:0015886	heme transport	1	0.05726	-1.162340103	Dinc136461-RA	-1.162340103
BP	GO:1902356	oxaloacetate(2-) transmembrane transport	1	0.05726	0.4987257115	Dinc118144-RA	0.4987257115
BP	GO:0010275	NAD(P)H dehydrogenase complex assembly	1	0.05726	0.6431243551	Dinc095068-RA	0.6431243551
BP	GO:0046686	response to cadmium ion	4	0.05765	1.641254793	Dinc061226-RA	0.6639482574
BP	GO:0046686	response to cadmium ion	4	0.05765	1.641254793	Dinc095511-RA	1.206590806
BP	GO:0046686	response to cadmium ion	4	0.05765	1.641254793	Dinc100816-RA	0.8893823495

BP	GO:0046686	response to cadmium ion	4	0.05765	1.641254793	Dinc114607-RA	0.522588174
BP	GO:0098542	defense response to other organism	8	0.06988	-0.1825017046	Dinc069517-RA	0.6598007837
BP	GO:0098542	defense response to other organism	8	0.06988	-0.1825017046	Dinc071342-RA	1.020382125
BP	GO:0098542	defense response to other organism	8	0.06988	-0.1825017046	Dinc098792-RA	-0.6373337471
BP	GO:0098542	defense response to other organism	8	0.06988	-0.1825017046	Dinc110851-RA	1.001190761
BP	GO:0098542	defense response to other organism	8	0.06988	-0.1825017046	Dinc117592-RA	0.6593527247
BP	GO:0098542	defense response to other organism	8	0.06988	-0.1825017046	Dinc118741-RA	-1.196891149
BP	GO:0098542	defense response to other organism	8	0.06988	-0.1825017046	Dinc119051-RA	-0.8121168586
BP	GO:0098542	defense response to other organism	8	0.06988	-0.1825017046	Dinc46235-RA	-1.210577412
BP	GO:0007623	circadian rhythm	3	0.07079	0.5232913243	Dinc096040-RA	0.8422094053
BP	GO:0007623	circadian rhythm	3	0.07079	0.5232913243	Dinc099695-RA	-0.825224594
BP	GO:0007623	circadian rhythm	3	0.07079	0.5232913243	Dinc100816-RA	0.8893823495
BP	GO:0051259	protein complex oligomerization	2	0.07101	-2.488638226	Dinc083258-RA	-1.336562116
BP	GO:0051259	protein complex oligomerization	2	0.07101	-2.488638226	Dinc092213-RA	-2.182903815
BP	GO:0071423	malate transmembrane transport	1	0.07105	0.4987257115	Dinc118144-RA	0.4987257115
BP	GO:2000033	regulation of seed dormancy process	1	0.07105	-0.7478107115	Dinc125403-RA	-0.7478107115
BP	GO:0002237	response to molecule of bacterial origin	1	0.07105	0.6593527247	Dinc117592-RA	0.6593527247
BP	GO:0051209	release of sequestered calcium ion into ...	1	0.07105	0.7752569184	Dinc133819-RA	0.7752569184
BP	GO:0009969	xyloglucan biosynthetic process	1	0.07105	1.145204752	Dinc068362-RA	1.145204752
BP	GO:0006121	mitochondrial electron transport, succin...	1	0.07105	-0.6261377154	Dinc097557-RA	-0.6261377154
BP	GO:0009099	valine biosynthetic process	1	0.07105	0.8267448131	Dinc114258-RA	0.8267448131
BP	GO:0042908	xenobiotic transport	3	0.07778	0.386376198	Dinc064770-RA	0.71099676
BP	GO:0042908	xenobiotic transport	3	0.07778	0.386376198	Dinc079444-RA	-0.8426623786
BP	GO:0042908	xenobiotic transport	3	0.07778	0.386376198	Dinc095461-RA	0.8008888244
BP	GO:0009767	photosynthetic electron transport chain	6	0.07845	1.379952116	Dinc069517-RA	0.6598007837

MOLECULAR ECOLOGY

BP	GO:0009767	photosynthetic electron transport chain	6	0.07845	1.379952116	Dinc071434-RA	0.7593059306
BP	GO:0009767	photosynthetic electron transport chain	6	0.07845	1.379952116	Dinc123050-RA	0.7282238924
BP	GO:0009767	photosynthetic electron transport chain	6	0.07845	1.379952116	Dinc129156-RA	0.8620306135
BP	GO:0009767	photosynthetic electron transport chain	6	0.07845	1.379952116	Dinc136090-RA	1.050426387
BP	GO:0009767	photosynthetic electron transport chain	6	0.07845	1.379952116	Dinc48160-RA	-0.6796090537
BP	GO:0010411	xyloglucan metabolic process	3	0.07915	3.573143325	Dinc063696-RA	1.825279683
BP	GO:0010411	xyloglucan metabolic process	3	0.07915	3.573143325	Dinc068362-RA	1.145204752
BP	GO:0010411	xyloglucan metabolic process	3	0.07915	3.573143325	Dinc41693-RA	3.218381347
BP	GO:0010325	raffinose family oligosaccharide biosynt...	1	0.08465	-0.533938237	Dinc088854-RA	-0.533938237
BP	GO:0010332	response to gamma radiation	1	0.08465	-0.9681321423	Dinc131512-RA	-0.9681321423
BP	GO:0042549	photosystem II stabilization	1	0.08465	0.7608185282	Dinc129215-RA	0.7608185282
BP	GO:0050482	arachidonic acid secretion	1	0.08465	-0.8096388295	Dinc47347-RA	-0.8096388295
BP	GO:0016998	cell wall macromolecule catabolic proces...	1	0.08465	0.5695894826	Dinc24444-RA	0.5695894826
BP	GO:0031222	arabinan catabolic process	1	0.08465	1.780601425	Dinc067761-RA	1.780601425
BP	GO:0009639	response to red or far red light	3	0.08803	-0.4382330863	Dinc066269-RA	0.6508601416
BP	GO:0009639	response to red or far red light	3	0.08803	-0.4382330863	Dinc078468-RA	-0.6073328114
BP	GO:0009639	response to red or far red light	3	0.08803	-0.4382330863	Dinc088481-RA	-0.8025693012
BP	GO:0009690	cytokinin metabolic process	2	0.09268	-0.08770499312	Dinc100816-RA	0.8893823495
BP	GO:0009690	cytokinin metabolic process	2	0.09268	-0.08770499312	Dinc134844-RA	-1.01341594
BP	GO:0006511	ubiquitin-dependent protein catabolic pr...	10	0.09354	-0.6714626081	Dinc061521-RA	-0.6338325901
BP	GO:0006511	ubiquitin-dependent protein catabolic pr...	10	0.09354	-0.6714626081	Dinc084164-RA	1.440906998
BP	GO:0006511	ubiquitin-dependent protein catabolic pr...	10	0.09354	-0.6714626081	Dinc090335-RA	-0.9481384862
BP	GO:0006511	ubiquitin-dependent protein catabolic pr...	10	0.09354	-0.6714626081	Dinc100547-RA	-0.7499962021
BP	GO:0006511	ubiquitin-dependent protein catabolic pr...	10	0.09354	-0.6714626081	Dinc115202-RA	-0.885508535
BP	GO:0006511	ubiquitin-dependent protein catabolic pr...	10	0.09354	-0.6714626081	Dinc118352-RA	0.8452304001

BP	GO:0006511	ubiquitin-dependent protein catabolic pr...	10	0.09354	-0.6714626081	Dinc123280-RA	0.8795492698
BP	GO:0006511	ubiquitin-dependent protein catabolic pr...	10	0.09354	-0.6714626081	Dinc134023-RA	-1.082270644
BP	GO:0006511	ubiquitin-dependent protein catabolic pr...	10	0.09354	-0.6714626081	Dinc28065-RA	-0.4353784662
BP	GO:0006511	ubiquitin-dependent protein catabolic pr...	10	0.09354	-0.6714626081	Dinc40329-RA	-0.5539129497
BP	GO:0045454	cell redox homeostasis	6	0.0938	2.620648732	Dinc082486-RA	2.109161296
BP	GO:0045454	cell redox homeostasis	6	0.0938	2.620648732	Dinc091072-RA	0.7439200922
BP	GO:0045454	cell redox homeostasis	6	0.0938	2.620648732	Dinc106626-RA	0.7186813697
BP	GO:0045454	cell redox homeostasis	6	0.0938	2.620648732	Dinc111345-RA	1.233519339
BP	GO:0045454	cell redox homeostasis	6	0.0938	2.620648732	Dinc114251-RA	0.978108282
BP	GO:0045454	cell redox homeostasis	6	0.0938	2.620648732	Dinc137274-RA	0.6358618101
BP	GO:0042742	defense response to bacterium	4	0.09405	-0.2575356937	Dinc069517-RA	0.6598007837
BP	GO:0042742	defense response to bacterium	4	0.09405	-0.2575356937	Dinc098792-RA	-0.6373337471
BP	GO:0042742	defense response to bacterium	4	0.09405	-0.2575356937	Dinc117592-RA	0.6593527247
BP	GO:0042742	defense response to bacterium	4	0.09405	-0.2575356937	Dinc118741-RA	-1.196891149
BP	GO:0009833	plant-type primary cell wall biogenesis	2	0.09721	1.121887619	Dinc083646-RA	0.7897796325
BP	GO:0009833	plant-type primary cell wall biogenesis	2	0.09721	1.121887619	Dinc38121-RA	0.7968090533
BP	GO:0006099	tricarboxylic acid cycle	3	0.09745	-1.212974301	Dinc076296-RA	-0.7169375149
BP	GO:0006099	tricarboxylic acid cycle	3	0.09745	-1.212974301	Dinc097557-RA	-0.6261377154
BP	GO:0006099	tricarboxylic acid cycle	3	0.09745	-1.212974301	Dinc123601-RA	-0.7578578865
BP	GO:0046834	lipid phosphorylation	4	0.09751	-0.9185269393	Dinc074902-RA	0.927299539
BP	GO:0046834	lipid phosphorylation	4	0.09751	-0.9185269393	Dinc096279-RA	-1.405007851
BP	GO:0046834	lipid phosphorylation	4	0.09751	-0.9185269393	Dinc098675-RA	-0.5653950227
BP	GO:0046834	lipid phosphorylation	4	0.09751	-0.9185269393	Dinc100169-RA	-0.7939505437
BP	GO:0009052	pentose-phosphate shunt, non-oxidative b...	1	0.09804	0.4406984246	Dinc119830-RA	0.4406984246
BP	GO:0019563	glycerol catabolic process	1	0.09804	0.6593527247	Dinc117592-RA	0.6593527247

MOLECULAR ECOLOGY

BP	GO:1904482	cellular response to tetrahydrofolate	1	0.09804	-0.9940910615	Dinc061054-RA	-0.9940910615
BP	GO:1901401	regulation of tetrapyrrole metabolic pro...	1	0.09804	0.6122401898	Dinc093958-RA	0.6122401898
BP	GO:0006878	cellular copper ion homeostasis	1	0.09804	-0.7713420403	Dinc091616-RA	-0.7713420403
BP	GO:0006565	L-serine catabolic process	1	0.09804	-0.9940910615	Dinc061054-RA	-0.9940910615
BP	GO:0010032	meiotic chromosome condensation	1	0.09804	-0.9307413852	Dinc115014-RA	-0.9307413852
BP	GO:0009816	defense response to bacterium, incompati...	1	0.09804	-0.6373337471	Dinc098792-RA	-0.6373337471
BP	GO:0045040	protein import into mitochondrial outer ...	1	0.09804	0.8055403613	Dinc095941-RA	0.8055403613
BP	GO:0009938	negative regulation of gibberellic acid ...	1	0.09804	-0.7478107115	Dinc125403-RA	-0.7478107115
BP	GO:0006032	chitin catabolic process	1	0.09804	0.5695894826	Dinc24444-RA	0.5695894826

MOLECULAR ECOLOGY

Table S4. Linear mixed model fitting and significance results for various measurements obtained with MicroSpec v-1.0. Photosynthesis measurements were fitted with individuals, time, and date of the measurements modeled as random effects and species modeled as a fixed variable. All measurements were done for populations growing in the Alps in an interval of three days.

```
########## "Ambient_Humidity" ##########
Data: dtFspecMT
Models:
lmm.null: get(d) ~ 1 + (1 | Time_of_Day) + (1 | sample_name) + (1 | date)
lmm.full: get(d) ~ species + (1 | Time_of_Day) + (1 | sample_name) + (1 |
lmm.full:     date)
         Df    AIC    BIC  logLik deviance  Chisq Chi Df Pr(>Chisq)
lmm.null  5 422,30 434,14 -206,15   412,30
lmm.full  6 416,25 430,46 -202,12   404,25 8,0484      1   0,004554 **
---
Signif. codes:  0    ***    0,001    **    0,01    *    0,05    .    0,1         1
---------- SUMMARY ----------
Linear mixed model fit by maximum likelihood  ['lmerMod']
Formula: get(d) ~ species + (1 | Time_of_Day) + (1 | sample_name) + (1 |      date)
   Data: dtFspecMT

     AIC      BIC   logLik deviance df.resid
   416.2    430.5   -202.1    404.2       73

Scaled residuals:
     Min       1Q   Median       3Q      Max
-2.13244 -0.34430 -0.00416  0.33198  2.87213

Random effects:
 Groups      Name        Variance Std.Dev.
 sample_name (Intercept)  8.040   2.835
 Time_of_Day (Intercept)  5.356   2.314
 date        (Intercept) 53.107   7.287
 Residual                 2.496   1.580
Number of obs: 79, groups:  sample_name, 46; Time_of_Day, 7; date, 4

Fixed effects:
                     Estimate Std. Error t value
(Intercept)            56.181      3.862  14.546
speciestraunsteineri    3.467      1.129   3.072

Correlation of Fixed Effects:
            (Intr)
spcstrnstnr -0.188

########## "Ambient_Temperature" ##########
Data: dtFspecMT
Models:
lmm.null: get(d) ~ 1 + (1 | Time_of_Day) + (1 | sample_name) + (1 | date)
lmm.full: get(d) ~ species + (1 | Time_of_Day) + (1 | sample_name) + (1 |
lmm.full:     date)
         Df    AIC    BIC  logLik deviance  Chisq Chi Df Pr(>Chisq)
lmm.null  5 279,93 291,78 -134,97   269,93
lmm.full  6 241,47 255,68 -114,73   229,47 40,468      1  1,999e-10 ***
---
Signif. codes:  0    ***    0,001    **    0,01    *    0,05    .    0,1         1
---------- SUMMARY ----------
Linear mixed model fit by maximum likelihood  ['lmerMod']
Formula: get(d) ~ species + (1 | Time_of_Day) + (1 | sample_name) + (1 |      date)
   Data: dtFspecMT

     AIC      BIC   logLik deviance df.resid
   241.5    255.7   -114.7    229.5       73

Scaled residuals:
     Min       1Q   Median       3Q      Max
-2.18307 -0.34817  0.00108  0.32716  1.96516

Random effects:
 Groups      Name        Variance Std.Dev.
 sample_name (Intercept)  1.0920  1.0450
 Time_of_Day (Intercept) 14.6324  3.8252
 date        (Intercept)  3.8794  1.9696
 Residual                 0.1486  0.3855
Number of obs: 79, groups:  sample_name, 46; Time_of_Day, 7; date, 4

Fixed effects:
                     Estimate Std. Error t value
(Intercept)           25.9381     1.7891  14.497
speciestraunsteineri   4.0771     0.4406   9.253

Correlation of Fixed Effects:
            (Intr)
spcstrnstnr -0.159
```

Table S4. Continued.

```
########## "Leaf_Angle" ##########
Data: dtFspecMT
Models:
lmm.null: get(d) ~ 1 + (1 | Time_of_Day) + (1 | sample_name) + (1 | date)
lmm.full: get(d) ~ species + (1 | Time_of_Day) + (1 | sample_name) + (1 |
lmm.full:     date)
         Df    AIC    BIC  logLik deviance  Chisq Chi Df Pr(>Chisq)
lmm.null  5 700,37 712,22 -345,19   690,37
lmm.full  6 702,31 716,53 -345,16   690,31 0,0626      1     0,8024

########## "Leaf.Temp.Differential" ##########
Data: dtFspecMT
Models:
lmm.null: get(d) ~ 1 + (1 | Time_of_Day) + (1 | sample_name) + (1 | date)
lmm.full: get(d) ~ species + (1 | Time_of_Day) + (1 | sample_name) + (1 |
lmm.full:     date)
         Df    AIC    BIC  logLik deviance  Chisq Chi Df Pr(>Chisq)
lmm.null  5 205,01 216,86 -97,506   195,01
lmm.full  6 206,94 221,16 -97,472   194,94 0,0679      1     0,7944

########## "LEF" ##########
Data: dtFspecMT
Models:
lmm.null: get(d) ~ 1 + (1 | Time_of_Day) + (1 | sample_name) + (1 | date)
lmm.full: get(d) ~ species + (1 | Time_of_Day) + (1 | sample_name) + (1 |
lmm.full:     date)
         Df    AIC    BIC  logLik deviance  Chisq Chi Df Pr(>Chisq)
lmm.null  5 682,50 694,35 -336,25   672,50
lmm.full  6 681,45 695,67 -334,73   669,45 3,0467      1     0,0809 .
---
Signif. codes:  0    ***    0,001    **    0,01    *    0,05    .    0,1         1

########## "PAR" ##########
Data: dtFspecMT
Models:
lmm.null: get(d) ~ 1 + (1 | Time_of_Day) + (1 | sample_name) + (1 | date)
lmm.full: get(d) ~ species + (1 | Time_of_Day) + (1 | sample_name) + (1 |
lmm.full:     date)
         Df    AIC    BIC  logLik deviance  Chisq Chi Df Pr(>Chisq)
lmm.null  5 987,27 999,11 -488,63   977,27
lmm.full  6 985,10 999,32 -486,55   973,10 4,1642      1    0,04129 *
---
Signif. codes:  0    ***    0,001    **    0,01    *    0,05    .    0,1         1
---------- SUMMARY ----------
Linear mixed model fit by maximum likelihood  ['lmerMod']
Formula: get(d) ~ species + (1 | Time_of_Day) + (1 | sample_name) + (1 |      date)
   Data: dtFspecMT

     AIC      BIC   logLik deviance df.resid
   985.1    999.3   -486.6    973.1       73

Scaled residuals:
    Min      1Q  Median      3Q     Max
-1.8419 -0.4497 -0.1083  0.3832  1.9450

Random effects:
 Groups      Name        Variance Std.Dev.
 sample_name (Intercept) 10062    100.31
 Time_of_Day (Intercept)  2080     45.61
 date        (Intercept)  1241     35.23
 Residual                 5200     72.11
Number of obs: 79, groups:  sample_name, 46; Time_of_Day, 7; date, 4

Fixed effects:
                     Estimate Std. Error t value
(Intercept)            244.71      39.75   6.156
speciestraunsteineri    82.00      38.66   2.121

Correlation of Fixed Effects:
            (Intr)
spcstrnstnr -0.624
```

MOLECULAR ECOLOGY

Table S4. Continued.

```
########## "NPQt" ##########
Data: dtFspecMT
Models:
lmm.null: get(d) ~ 1 + (1 | Time_of_Day) + (1 | sample_name) + (1 | date)
lmm.full: get(d) ~ species + (1 | Time_of_Day) + (1 | sample_name) + (1 |
lmm.full:     date)
         Df    AIC    BIC  logLik deviance  Chisq Chi Df Pr(>Chisq)
lmm.null  5 84,801 96,649 -37,401   74,801
lmm.full  6 80,908 95,124 -34,454   68,908 5,8937      1    0,01519 *
---
Signif. codes:  0    ***    0,001    **    0,01    *    0,05    .    0,1         1
---------- SUMMARY ----------
Linear mixed model fit by maximum likelihood  ['lmerMod']
Formula: get(d) ~ species + (1 | Time_of_Day) + (1 | sample_name) + (1 |      date)
   Data: dtFspecMT

     AIC      BIC   logLik deviance df.resid
    80.9     95.1    -34.5     68.9       73

Scaled residuals:
    Min      1Q  Median      3Q     Max
-1.4270 -0.4594 -0.1077  0.3824  1.9979

Random effects:
 Groups      Name        Variance Std.Dev.
 sample_name (Intercept) 0.1495   0.3867
 Time_of_Day (Intercept) 0.0000   0.0000
 date        (Intercept) 0.1017   0.3188
 Residual                0.0404   0.2010
Number of obs: 79, groups:  sample_name, 46; Time_of_Day, 7; date, 4

Fixed effects:
                     Estimate Std. Error t value
(Intercept)            0.7280     0.1948   3.737
speciestraunsteineri   0.3428     0.1327   2.584

Correlation of Fixed Effects:
            (Intr)
spcstrnstnr -0.460

########## "Phi2" ##########
Data: dtFspecMT
Models:
lmm.null: get(d) ~ 1 + (1 | Time_of_Day) + (1 | sample_name) + (1 | date)
lmm.full: get(d) ~ species + (1 | Time_of_Day) + (1 | sample_name) + (1 |
lmm.full:     date)
         Df     AIC     BIC logLik deviance  Chisq Chi Df Pr(>Chisq)
lmm.null  5 -224,16 -212,31 117,08  -234,16
lmm.full  6 -231,92 -217,70 121,96  -243,92 9,7598      1   0,001784 **
---
Signif. codes:  0    ***    0,001    **    0,01    *    0,05    .    0,1         1
---------- SUMMARY ----------
Linear mixed model fit by maximum likelihood  ['lmerMod']
Formula: get(d) ~ species + (1 | Time_of_Day) + (1 | sample_name) + (1 |      date)
   Data: dtFspecMT

     AIC      BIC   logLik deviance df.resid
  -231.9   -217.7    122.0   -243.9       73

Scaled residuals:
    Min      1Q  Median      3Q     Max
-2.0848 -0.4408 -0.0179  0.5920  1.4665

Random effects:
 Groups      Name        Variance  Std.Dev.
 sample_name (Intercept) 2.152e-03 4.639e-02
 Time_of_Day (Intercept) 4.577e-04 2.139e-02
 date        (Intercept) 2.084e-18 1.444e-09
 Residual                1.065e-03 3.263e-02
Number of obs: 79, groups:  sample_name, 46; Time_of_Day, 7; date, 4

Fixed effects:
                     Estimate Std. Error t value
(Intercept)           0.48411    0.01618   29.93
speciestraunsteineri -0.05933    0.01771   -3.35

Correlation of Fixed Effects:
            (Intr)
spcstrnstnr -0.694
```

MOLECULAR ECOLOGY

Table S4. Continued.

```
########## "PhiNO" ##########
Data: dtFspecMT
Models:
lmm.null: get(d) ~ 1 + (1 | Time_of_Day) + (1 | sample_name) + (1 | date)
lmm.full: get(d) ~ species + (1 | Time_of_Day) + (1 | sample_name) + (1 |
lmm.full:     date)
         Df     AIC     BIC logLik deviance  Chisq Chi Df Pr(>Chisq)
lmm.null  5 -236,00 -224,15 123,00  -246,00
lmm.full  6 -236,27 -222,05 124,13  -248,27 2,2715      1     0,1318

########## "PhiNPQ" ##########
Data: dtFspecMT
Models:
lmm.null: get(d) ~ 1 + (1 | Time_of_Day) + (1 | sample_name) + (1 | date)
lmm.full: get(d) ~ species + (1 | Time_of_Day) + (1 | sample_name) + (1 |
lmm.full:     date)
         Df     AIC     BIC logLik deviance  Chisq Chi Df Pr(>Chisq)
lmm.null  5 -198,11 -186,26 104,05  -208,11
lmm.full  6 -204,95 -190,74 108,48  -216,95 8,8481      1   0,002934 **
---
Signif. codes:  0    ***    0,001    **    0,01    *    0,05    .    0,1        1
---------- SUMMARY ----------
Linear mixed model fit by maximum likelihood  ['lmerMod']
Formula: get(d) ~ species + (1 | Time_of_Day) + (1 | sample_name) + (1 |      date)
   Data: dtFspecMT

     AIC      BIC   logLik deviance df.resid
  -205.0   -190.7    108.5   -217.0       73

Scaled residuals:
    Min      1Q  Median      3Q     Max
-2.2984 -0.4329 -0.1064  0.4454  2.1185

Random effects:
 Groups      Name        Variance  Std.Dev.
 sample_name (Intercept) 0.0038732 0.06223
 Time_of_Day (Intercept) 0.0009791 0.03129
 date        (Intercept) 0.0008445 0.02906
 Residual                0.0010926 0.03305
Number of obs: 79, groups:  sample_name, 46; Time_of_Day, 7; date, 4

Fixed effects:
                     Estimate Std. Error t value
(Intercept)           0.21119    0.02647   7.977
speciestraunsteineri  0.07903    0.02302   3.433

Correlation of Fixed Effects:
            (Intr)
spcstrnstnr -0.559
```

MOLECULAR ECOLOGY

Table S4. Continued.

```
########## "Relative_Chlorophyll" ##########
Data: dtFspecMT
Models:
lmm.null: get(d) ~ 1 + (1 | Time_of_Day) + (1 | sample_name) + (1 | date)
lmm.full: get(d) ~ species + (1 | Time_of_Day) + (1 | sample_name) + (1 |
lmm.full:     date)
         Df    AIC    BIC  logLik deviance  Chisq Chi Df Pr(>Chisq)
lmm.null  5 541,90 553,75 -265,95   531,90
lmm.full  6 537,79 552,01 -262,90   525,79 6,1055      1    0,01348 *
---
Signif. codes:  0    ***    0,001    **    0,01    *    0,05    .    0,1        1
---------- SUMMARY ----------
Linear mixed model fit by maximum likelihood  ['lmerMod']
Formula: get(d) ~ species + (1 | Time_of_Day) + (1 | sample_name) + (1 |      date)
   Data: dtFspecMT

     AIC      BIC   logLik deviance df.resid
   537.8    552.0   -262.9    525.8       73

Scaled residuals:
     Min       1Q   Median       3Q      Max
-1.92730 -0.43289 -0.03472  0.46551  2.17496

Random effects:
 Groups      Name        Variance Std.Dev.
 sample_name (Intercept) 31.2130  5.5869
 Time_of_Day (Intercept)  0.0000  0.0000
 date        (Intercept)  0.1999  0.4471
 Residual                22.6397  4.7581
Number of obs: 79, groups:  sample_name, 46; Time_of_Day, 7; date, 4

Fixed effects:
                     Estimate Std. Error t value
(Intercept)            42.931      1.646  26.088
speciestraunsteineri   -5.327      2.053  -2.595

Correlation of Fixed Effects:
            (Intr)
spcstrnstnr -0.784
```
